# Biomaterials-based additive manufacturing for customized bioengineering in management of otolaryngology: a comprehensive review

**DOI:** 10.3389/fbioe.2023.1234340

**Published:** 2023-09-08

**Authors:** Jigar Vyas, Isha Shah, Sudarshan Singh, Bhupendra G. Prajapati

**Affiliations:** ^1^ Sigma Institute of Pharmacy, Vadodara, Gujarat, India; ^2^ Department of Pharmaceutical Sciences, Faculty of Pharmacy, Chiang Mai University, Chiang Mai, Thailand; ^3^ Office of Research Administration, Chiang Mai University, Chiang Mai, Thailand; ^4^ Shree S. K. Patel College of Pharmaceutical Education and Research, Ganpat University, Kherva, India

**Keywords:** otolaryngology, three dimensional printing, four-dimensional printing, additive manufacturing, biomaterials, bioprinting

## Abstract

Three-dimensional (3D)/four-dimensional (4D) printing, also known as additive manufacturing or fast prototyping, is a manufacturing technique that uses a digital model to generate a 3D/4D solid product. The usage of biomaterials with 3D/4D printers in the pharma and healthcare industries is gaining significant popularity. 3D printing has mostly been employed in the domain of otolaryngology to build portable anatomical models, personalized patient-centric implants, biologic tissue scaffolds, surgical planning in individuals with challenging conditions, and surgical training. Although identical to 3D printing technology in this application, 4D printing technology comprises a fourth dimension of time. With the use of 4D printing, a printed structure may alter over time under various stimuli. Smart polymeric materials are also generally denoted as bioinks are frequently employed in tissue engineering applications of 3D/4D printing. In general, 4D printing could significantly improve the safety and efficacy of otolaryngology therapies. The use of bioprinting in otolaryngology has an opportunity to transform the treatment of diseases influencing the ear, nose, and throat as well as the field of tissue regeneration. The present review briefs on polymeric material including biomaterials and cells used in the manufacturing of patient centric 3D/4D bio-printed products utilized in management of otolaryngology.

## Graphical Abstract

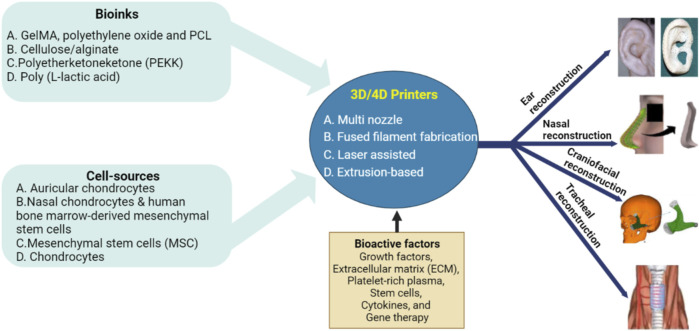

## 1 Introduction

3D printing, also known as additive manufacturing (AM) or rapid prototyping, is a manufacturing technique that uses a 3D digital model to fabricate a 3D object. It was first developed by Charles Hull in the early 1980s, and demand for the process has grown significantly since then (Ventola, 2014). It is a fast and efficient way to turn computer designs into actual objects (Hoy, 2013). Digital 3D models are often developed using computer-aided design software (CAD) or retrieved using 3D scanners that take photos and dimensions of actual products before sending the information to a computer. The use of 3D printers in the pharmaceutical and healthcare industries is becoming increasingly popular. This technology is being used in the medical field for the development of personalized medicines, oral dosage forms, medical devices, and tissue engineering applications (Goyanes et al., 2016). A 3D-printed product often consists of numerous thin layers of material placed on top of each other. For regenerative therapies, 3D bioprinting has been used to produce medical implants or scaffolds for 3D-printed cells (Cui et al., 2017). 3D printing in otolaryngology is used for planning complex surgeries, training surgeons, and replacing missing tissue. Additionally, 3D printing has been used in the field of otolaryngology primarily for the fabrication of wearable anatomical models, personalized patient-specific implants, biological tissue scaffolds, surgical planning in individuals with challenging conditions, and surgical education (Kaye et al., 2016a).

Bioengineering based on biomaterials has transformed the field of head and neck surgery. Bone graft implantation is one of the most common applications of biomaterials in head and neck surgery. Bone grafts are used to support dental implants or to restore lost jawbones. These grafts are often made from synthetic materials such as hydroxyapatite or from natural elements such as bone from another region of the patient’s body. Another important application of biomaterials in head and neck surgery is the formation of tissue engineered structures. Tissue engineering involves designing 3D structures that promote the development of new tissue. Moreover, biomaterials are also used in the manufacture of implants and prostheses. These devices are used to repair damaged structures, including the ear or larynx. With implants and prostheses made using biomaterials are tailored to replicate the function and structure of the original tissue, patients can restore their normal function and appearance (Gualtieri et al., 2021).

3D printing technology has seen significant improvements in materials, devices, and processes capable of revolutionizing everything from our daily lives to the global system. Although identical to 3D printing technology in this application, 4D printing technology encompasses a fourth dimension of time. Using 4D printing, a printed structure can change over time in response to external stimuli. Temperature, humidity, pH, light, pressure, and magnetic fields are all examples of stimuli. From tissue engineering to the development of self-assembled biomaterials on a human scale, 4D printing is useful for a wide range of therapeutic purposes (Shim et al., 2012; Pati et al., 2014). Personalized medicine, smart pharmacology, and programmable cells and tissues used to target diseases could be enabled by the ability of this technology to develop customizable biological materials with modifiable shapes and properties (Shim et al., 2012). For the development of stimuli-responsive behaviors, 4D printing requires smart polymeric materials with advanced properties. Moreover, dielectric elastomers, shape memory polymers, stimuli-responsive hydrogels, shape memory metal alloys, and smart nanocomposites have been used in 4D printing (Zhao et al., 2015). In another study, Kim and co-workers developed a cell-friendly and biocompatible 4D bioprinting system including more than 2 cell types based on digital light processing and photocurable silk fibroin hydrogel. The shape changes of 3D printed bilayer silk fibroin hydrogels were controlled by modulating their interior or exterior properties in physiological conditions. Though implants were integrated with the host trachea naturally, and both epithelium and cartilage were formed at the predicted sites, the tissue mimetic properties were required to be assessed (Kim et al., 2020b).

The fabrication of individualized implants that change shape to adapt fluctuating structure of the body is one of the most important applications of 4D printing in otolaryngology, especially in sensitive regions such as the ear, nose, and throat. The 3D/4D bioprinting has the potential to address and circumvent current limitations in reconstructive surgery including the need for donor tissue, poor tissue match, and transplant rejection. The rapid growth of 3D/4D printing technologies may allow for anatomically accurate reconstructive options to generate tissues of head and neck, notably musculoskeletal tissue such as cartilage and bone. In general, 4D printing significantly improves the safety and efficacy of therapies in otolaryngology. While 3D/4D bioprinting holds great promise in various medical fields, including otolaryngology conditions there are some limitations such as complexity of tissue structures, vascularization and innervation, material selection, long-term stability and integration, and most important regulatory consideration. However, to promote the future of management otolaryngology using 3D/4D bioprinting, overcoming these limitations requires, technological advancements, and collaboration between experts in the field. Therefore, the present review provides an overview of various polymeric materials, including biomaterials and cells, for the fabrication of patient-centred 3D/4D bioprinting products in management of otolaryngology.

## 2 Printable bioinks

Bioinks are biocompatible materials such as hydrogels or polymers that serve as scaffolds for cell growth and differentiation to form functional tissue. By adding cells and growth factors, these bioinks are tailored to mimic the properties of real tissue, such as cartilage, bone, or skin, and contribute to tissue regeneration. Moreover, bioinks are stimulus-responsive materials that change shape or functional properties in response to external stimuli. Depending on the type of stimuli used to activate the 4D process, these bioinks are classified as chemical, physical, or biological stimuli-responsive materials. The combination of AM with bioinks led to the development of a new field of research, 3D/4D printing (Zhou et al., 2015). Compared to conventional AM methods, 3D/4D printing offers new perspectives for the fabrication of smart or dynamic devices and structures instead of static structures. Table 1 illustrates summarizes stimuli-responsive biomaterials for tissue engineering by adopting advanced technology.

**TABLE 1:** Stimuli-responsive materials with their applications.

Stimulus	Bioinks used for 4DP	Stimuli	Application	References
*Chemical stimuli-responsive materials*				
Ion-sensitive	Poly (acrylonitrile)	Crosslinked with multivalent ions (Zn^2+^ and Ca^2+^)	Fabrication of cell-loaded, shape-changing objects for tissue engineering	Bai et al. (2012)
pH-responsive	Poly (N-isopropylacrylamide)	Shape transition in pH range (pH 2–10)	Controlled release of drugs, cell encapsulation, tissue engineering	Nadgorny and Ameli (2018)
	Chitosan/TPP	Shape transition in pH range (pH 4–7)	Regenerative bone medicine	Xu et al. (2018)
***Physical stimuli-responsive materials***				
Temperature-responsive	Poly (caprolactone) (PCL)	Change in hydrogel shape due to thermal activation at 37°C	Bone defects	Zarek et al. (2017)
Magnetic Responsive	Poly (lactic acid)	Change in hydrogel shape due to magnetic field (30 kHz)	Tissue engineering, drug delivery devices, and actuators	Wei et al. (2017)
Electro-responsive	Poly (thiophene), poly (aniline), and poly (pyrrole)	Electrical conduction causes hydrogel to change shape	Neuro-prosthetic devices and bioelectronic designs	Fantino et al. (2018)
Photo-responsive	Poly (lactic acid)	Change in shape using a UV cross-linking agent	Soft robotics, flexible electronics, minimally invasive medicine	Wei et al. (2017)
	Urethane diacrylate and a linear semicrystalline polymer		Soft actuators, deployable smart medical devices, and flexible electronics	Kuang et al. (2018)
Water-responsive	PCL, PEG, and cellulose nanocrystals (CNCs)	Water absorption leads to shape change	Self-tightening sutures and self-retractable smart stents	Li et al. (2015)
***Biological stimuli-responsive materials***				
Enzyme	Hyaluronic acid	Activation of shape memory capabilities of hydrogels	Tissue engineering by improving tissue defect regeneration and tissue remodelling	Wang (2018)

### 2.1 Chemical stimuli-responsive materials

Chemically stimulated materials are those that reversibly change their structure, properties, or behavior in response to chemical stimuli. These include ion-sensitive hydrogels and pH-responsive polymers.

#### 2.1.1 Ion-sensitive hydrogels

Several studies have shown that it is possible to fabricate scaffolds that withstand cell-filled structures on a clinical scale (Hockaday et al., 2012). To fabricate scaffolds with sufficient strength and versatile mechanical properties, bioprinting enables crosslinking with multivalent ions such as Zn^2+^ and Ca^2+^ (Tabriz et al., 2015). Bai et al. performed bidirectional dipole-dipole interactions of poly (acrylonitrile) chains in response to Zn^2+^ to develop a reversible shape memory hydrogel with ultra-high strength for cells (Bai et al., 2012). A novel shape memory hydrogel was developed by imidazole-zinc ion coupling (Nan et al., 2013). By using chelating chemicals to remove zinc ions, the stable shapes of these fabricated structures are perhaps restored, which exhibit bidirectional memory function. To maintain the flat hydrogel layer with cells, it is possible to press them into a temporary cylindrical shape and culture them with zinc ions. These ion-sensitive crosslinking hydrogels offer unique alternatives for the fabrication of cell-loaded, shape-changing objects in the context of tissue engineering and 4D bioprinting technologies.

#### 2.1.2 pH-responsive materials

Chemical groups that respond to pH, such as carboxyl, pyridine, sulfone, and phosphate groups, are explored to fabricate self-assembling structures such as pH-responsive substances capable of transition from spheres to spirals when pH adjusted to a certain range. The polymer chains in electrostatic repellent phases transform into globules when the functional group of the polymer poly (N-isopropylacrylamide) is neutralized (Nadgorny and Ameli, 2018). It is possible to prepare chitosan/TPP scaffolds with different cross-linking primary amine loading with protein for bone regenerative therapies. The adsorption and release behavior are pH-sensitive and is influenced by the degree of cross-linking of amine (Xu et al., 2018). The newly designed scaffolds with flexible shape and mechanical properties fabricated using pH-sensitive materials are biologically significant and capable of providing alternatives to 4D bioprinting.

### 2.2 Physical stimuli-responsive materials

Stimuli that respond to temperature changes, magnetic fields, electromagnetism, light, and moisture cause changes in the internal atomic packing configurations or chain dynamics of materials, resulting in shape-changing behavior. Polymeric materials with this type of shape-changing behavior have special properties that can adapt to physical changes in environmental conditions. This is an important consideration for their future use in biomedical applications.

#### 2.2.1 Temperature-responsive materials

The physical stimulus most commonly used to induce shape changes in biologically printed objects is temperature (Bakarich et al., 2015). Some examples of temperature-sensitive polymeric materials are poly (N-isopropylacrylamide), polyethylene glycol, chitosan, gelatin, and poly (caprolactone) (PCL). This allows the structure to transform into the desired shape upon application if sufficient transformation energy is available. Such shape changing properties could be very beneficial for self-fitting replacement implants for smaller bone defects. For instance, Zarek and colleagues used selective laser assisted (SLA) to develop a methacrylate-based tracheal stent that was thermally activated (Zarek et al., 2017). To avoid potential damage during the implantation procedure, the stent could expand and fit comfortably after implantation in the tracheal region of the body.

Apsite and colleagues prepared porous multi-layer scaffolds using thermo-responsive polymers poly (N-isopropylacrylamide) and PCL. The scaffolds spontaneously rolled into tubular structures with PCL as the inner layer in water-based conditions at 37°C, making them appropriate for cell encapsulation. When the polymers are covered with collagen, the cell adherence and viability are increased, which increases scaffold’s applicability for numerous tissue engineering needs (Apsite et al., 2017).

#### 2.2.2 Magnetic responsive materials

Wei et al. developed an ink of poly (lactic acid) polymer in which magnetic iron oxide nanoparticles are embedded to fabricate tubular constructs with magnetically controllable and recoverable shape properties using AM technology (Wei et al., 2017). The iron oxide was heated in the presence of a resistive magnetic field that generated sufficient force to return the temporary shape to its original form. Adjusting the force and orientation of the magnetic field during tissue engineering could lead to changes in scaffold morphology and geometry. This could be used in applications such as alignment of structural elements, application of mechanical stimulation, and stem cell differentiation. Due to their excellent rheological properties, hydrogels mimicked using a magnetic field offer advantageous opportunities for biomaterial printing (Sonatkar et al., 2022). In another study, Zhu et al. fabricated 4D-printed bioproducts responsive to magnetic fields by adding Fe-based NPs to poly (dimethylsiloxane). As part of the bioprinting process that develops scaffolds with anisotropic properties, the magnetized bio-prints perhaps help to regulate the direction of the NPs (Zhu et al., 2018).

#### 2.2.3 Electro-responsive materials

The major component of electro-responsive materials is polyelectrolytic polymers that expand, compress, or fold in response to an electric field. Both the direction and the strength of the electric field can affect the properties of these materials (Borisova et al., 2015). Some hydrogels enriched with electrically conductive polymers such as poly (thiophene), poly (aniline), and poly (pyrrole) exhibit good biocompatibility and printability, opening the way for the possibility of 4D printing. For example, 3D printing and polypyrrole interfacial polymerization have been found to form a conductive electroactive hydrogel (Fantino et al., 2018). The printed structures are used for the development of novel neuro-prosthetic devices and bioelectronic designs due to their distinct mechanical properties, advantageous electrical conductivity, and adaptable surface chemistry. Electro-responsive carbon-based nano-biomaterials such as carbon nanotubes and graphene have attracted great interest in the last decade as strategies to study and regulate stem cell physiology and fate (Ahadian et al., 2016). The use of electrically conductive and biocompatible carbon-based nano-biomaterials to develop stimuli-responsive 4D structures could enhance bone tissue and brain regeneration (Kai et al., 2016).

#### 2.2.4 Photo-responsive materials

Direct transmission of optical pulses can cause mechanical reactions in materials that respond to light. Near-infrared, infrared, and ultraviolet light bands activate photo-responsive biomaterials that are commonly used in biomedical disciplines such as tissue engineering and controlled drug release (Cui et al., 2019). The most common reaction pathways for photosensitive materials used extensively in the construction of active, shape changing 4D structures are photoisomerization and photodegradation of polymer chains. Wei et al. used a UV crosslinking agent to print a tubular poly (lactic acid) structure with shape memory (Wei et al., 2017), while Kuang et al. explored a UV light-assisted bioink composed of a linear semi-crystalline polymer and urethane diacrylate. The polymeric bioink exhibited excellent shape memory and self-healing properties to form a basis for the development of 4D bioprinting (Kuang et al., 2018). In another study, Arakawa et al. used customizable photodegradation of biomaterials to develop multicellular 3D endothelial vascular networks in cell-filled hydrogels. The multiphoton lithography approach with programmable 4D control enables rapid construction of networks of microchannels with diameters corresponding to real human vasculature. As a result, these photo-responsive 4D bioinks are capable of mimicking the dynamic features of extracellular matrix decay in real life (Arakawa et al., 2017).

#### 2.2.5 Water-responsive materials

The humidity transformation of moisture-responsive materials is stimulated by water. One strategy for humidity-responsive 4D printing of scaffolds is to employ a composite material composed of a hydrogel matrix embedded with humidity-responsive particles/fibers. This combination enables the scaffold to demonstrate regulated dimensional modifications in response to fluctuations in humidity levels. The most popular moisture-responsive materials utilized for 4D printing are poly (ethylene glycol) (PEG) and hydrogels. A humidity sensitive and thermo-responsive nanocomposite, fabricated from PCL, PEG, and cellulose nanocrystals (CNCs) nanofillers. When this formulated nanocomposite strip was immersed in 37°C water, it returned to its original shape due to its water absorption and swelling capacity (Li et al., 2015).

### 2.3 Biological stimuli-responsive materials

Bio-enzymes, glucose, and other biological stimuli are used in biological responsive materials (El-Husseiny et al., 2022). Some bio-enzymes cleave biomolecules and peptide chains, leading to changes in polymers and causing them to swell further. Shape memory hydrogels show ionic interactions with polypeptides, polynucleotides, or reversible hydrogen bonds in addition to gelation through ionic crosslinking. A 4D structure designed by encapsulating biologically active molecules such as enzymes and antibodies in 3D objects. Enzymes are crucial for the functioning of various biological reactions. Enzymes found in the human body are used to activate the shape memory capabilities of hydrogels. Hydrogels require enzyme substrates that act as crosslinkers or functional side groups (Wang, 2018). An important protease involved in the degradation of extracellular matrix components is matrix metalloproteinase. Hydrogels of hyaluronic acid that were sensitive to matrix metalloproteinase showed significant cell attachment and tunable swelling and degradation properties (Kim et al., 2008).

## 3 Cell sources

In the field of otolaryngology, tissue regeneration using 3D/4D printing has emerged as a potential strategy. For effective tissue regeneration, the right cell sources are an important request. Moreover, for tissue regeneration in otolaryngology using 3D/4D printing, excellent cell sources demand critical characteristics. First, such cells should be able to identify and proliferate the exact cell types required for tissue repair to ensure adequate regeneration. Because 3D/4D printing involves stringent conditions such as mechanical forces and bioink compositions with viability during printing. In addition, immune compatibility is another important feature, as cells selected might likelihood of immunological rejection in the recipient. Finally, reliable, and consistent results in tissue regeneration, differentiation, and proliferation are critical for the effective functioning of the printed structures. Considering these characteristics, researchers, and clinicians can select appropriate cell sources that meet the requirements of 3D/4D printing for optimal tissue regeneration in otolaryngology. Chondrocytes, osteoblasts, stem cells and epithelial cells are some types of such cell sources used in 3D/4D printing for tissue regeneration in otolaryngology.

Cells referred to as chondrocytes are responsible for the production and maintenance of the body’s cartilage tissue. Additionally, chondrocytes are used as a cell source in 3D printing for tissue regeneration in otolaryngology. They are commonly used to regenerate cartilage tissue in the nose or ears. Osteoblasts are the cells responsible for mineralizing and building bone. Osteoblasts are used as a cell source for 3D printing in otolaryngology to repair bone tissue in the nose or ears (Chiu et al., 2017). In addition, osteoblasts are the cells responsible for mineralization and construction of bone. In otolaryngology, osteoblasts are employed as a cell source for 3D printing to repair bone tissue of nose or ears (Adel-Khattab et al., 2018). Stem cells are undifferentiated cells that differentiate into a wide range of body cells. In the treatment of otolaryngology, stem cells may be employed as a cell source for 3D printing tissue regeneration. Induced pluripotent stem cells, adult stem cells, and embryonic stem cells are among the stem cell types that are utilized for 3D printing in otolaryngology. To assist their development and growth it needs scaffolding material. Hyaluronic acid, collagen, and synthetic polymers are examples of biodegradable materials that are frequently employed as scaffold materials in 3D printing for tissue regeneration (King et al., 2012). In addition to their capacity to develop the outer layer of many kinds of structures in the head and neck region, epithelial cells are a viable source for tissue regeneration in the otolaryngology area. The ear, nose, and throat’s epithelial tissues are essential to their health and disruption of them lead to a variety of ailments and diseases. The primary benefit of using epithelial cells for tissue regeneration is that they are easily extracted from the patient’s tissue, minimizing the risk of immune system reactions and rejection. For tissue engineering applications, epithelial cells are extracted from a variety of sources, including the patient’s skin or mucosal tissue, and cultured in laboratories. A variety of 3D/4D printing methods for epithelial tissues employing varied substances and cell sources are investigated. Among them, a technique to develop scaffolds for tissue formation by incorporating epithelial cells into a hydrogel-based biomaterial is widely explored. Moreover, 3D/4D printing of vocal fold tissues and nasal cartilage has gained significant attention among researchers. A method utilizing epithelial cells and a biodegradable polymer scaffold to produce a replacement for tracheal tissue in human patients had been proven to be effective in the future as indicated by experimental animal results (Park et al., 2019). Structures that significantly replicate actual cartilage tissue are developed with cell sources in 3D/4D printing. To assist their growth and development, it needs scaffolding material. In 3D/4D printing, a variety of biodegradable polymeric materials are frequently employed as scaffold materials for cartilage tissue regeneration. A promising method that might enhance the result of reconstructive procedures and minimize the requirement for synthetic implants or donor tissue is the application of a cell source for 3D/4D printing in tissue regeneration in otolaryngology.

## 4 Bioactive factors

In the area of otolaryngology, bioactive substances are essential as they stimulate tissue development, repair, and regeneration. Growth factors, extracellular matrix (ECM), platelet-rich plasma, stem cells, cytokines, and gene therapy are a few of the bioactive substances employed in otolaryngology. Moreover, growth factors are proteins that promote cell division, growth, and proliferation. Growth factors including platelet-derived growth factor, transforming growth factor-β, epidermal growth factor, and fibroblast growth factor are frequently utilized in otolaryngology to stimulate tissue repair and regeneration. Hyaluronic acid and collagen are two ECM components that act as a scaffold for cell adhesion and motility, which is essential for tissue regeneration. The elements of ECM have applications in otolaryngology to encourage tissue regeneration and wound healing (Brown and Badylak, 2014). Platelets and growth factors from the blood of the individual are concentrated to form platelet-rich plasma. It is frequently employed to treat ailments including chronic sinusitis, encourage regeneration, and speed up wound healing (Stavrakas et al., 2016). Undifferentiated cells with the potential to differentiate into tissue multiple cells are called stem cells. Due to their capacity to differentiate into bone, cartilage, and other connective tissues, mesenchymal stem cells (MSCs) are frequently utilized in otolaryngology for tissue regeneration (Kaboodkhani et al., 2021). Proteins called cytokines control immunological response and inflammation. In otolaryngology, cytokines such as tumor necrosis factor-α, interleukin-1, and interleukin-6 are employed to support tissue regeneration and repair (Zgheib et al., 2014). Gene therapy is the insertion of genes into cells to alleviate or cure illness by modifying the bioactive elements. Gene therapy is employed in otolaryngology to promote tissue regeneration (Hosseinkhani et al., 2023).

## 5 Current methods of 3D bioprinting

Bioprinting, also known as 3D bioprinting integrates AM and biotechnology principles to fabricate 3D/4D objects utilizing live cells, biochemical components, and polymeric biomaterials. By enabling accurate positioning of cells and materials to manufacture functional biological constructions, it serves as an important advancement in the field of regenerative medicine and tissue engineering. The common processes utilized in 3D/4D bioprinting are micro-extrusion, droplet 3D/4D bioprinting, and light-based 3D/4D bioprinting.

### 5.1 Micro extrusion

The extrusion-based bioprinting approach uses fluid-dispensing equipment and an automated robotic machine to extrude gel-form bioink and bio-print a model (Mironov et al., 2003) Printed objects develop 3D custom-shaped structures as a result of cylindrical filaments that could include biological components like cells. Polycaprolactone is often utilized for micro extrusion printing in the construction of scaffolds. A biodegradable polyester known as PCL has been utilized extensively in tissue engineering because of its biocompatibility and slow rate of disintegration. Other materials, including shape-memory polymers or hydrogels are also investigated with PCL to develop the scaffold smart or 4D printing characteristics. These smart components are combined with the PCL to produce a scaffold that changes over time, potentially serving as a tool for regenerative medicine and tissue engineering.

Extrusion-based bioprinters are both inexpensive as well as simple to assemble. High-viscosity biomaterials are utilized as bioink in the fabrication of small and large tissues or scaffolds that are unique to a certain tissue. Using cell spheroids or cell aggregates as bioink allows for the printing of complex tissues that self-assemble. These techniques have the problem of only being able to extrude highly viscous materials. Low-viscosity materials require high pressure for extrusion which leads to significant shear stress and a propensity for cell death.

### 5.2 Droplet 3D bioprinting

A customized inkjet printer is used to build 3D structures by depositing tiny droplets of bioink on a substrate or scaffold using the inkjet bioprinting technique (Öblom et al., 2020). Using heat, piezoelectric, or electrostatic forces, the printer dispenses the bioink, which is a hydrogel-based substance containing live cells, nutrition, and growth factors. A potential method to develop complicated, stimuli-responsive scaffolds for tissue engineering and regenerative medicine purposes is 4D printing utilizing inkjet bioprinting. Usually, a hydrogel-based bioink with living cells, nutrients, and growth factors is utilized in inkjet bioprinting for fabricating 3D/4D scaffolds. Alginate, collagen, gelatin, polyethylene glycol, hyaluronic acid, and polyvinyl alcohol are examples of the natural and synthetic polymeric biomaterials that are commonly found in hydrogel-based bioink. These materials are altered to produce the necessary porosity, stiffness, and degradation rate for the scaffold’s mechanical and biological qualities. To allow 4D printing, stimuli-responsive materials such as hydrogels or shape memory polymers may additionally be added to the scaffold design in addition to the hydrogel-based bioink (Saska et al., 2021). Droplet 3D printing offers several advantages, including high resolution and accuracy, adaptability, and scalability. The restricted variety of printable biomaterials and the possibility for decreased cell survival as a result of the significant shear stresses applied to the cells throughout printing are some of its drawbacks.

### 5.3 Light-based 3D bioprinting

Light assisted 3D/4D bioprinting is an advancement around bioprinting that uses light to start and direct the process of printing. It entails the application of photosensitive biomaterials that are referred to as bioinks, which solidify or crosslink when exposed to a certain light wavelength. This method enables fine spatial control over cell and biomaterial deposition, allowing the production of complex 3D/4D structures with great precision. The two main types of light-based 3D bioprinting are SLA and stereolithography.

#### 5.3.1 Selective laser assisted

Another popular 3D/4D printing method is SLA printing which employs light (laser) emitted at various frequencies for solidifying liquid resins and polymers. This technique uses lasers to layer by layer cure a liquid resin to develop a 3D/4D structure. After being dried, photopolymers transform into gels that cross-link to form solid polymers. It is frequently utilized for developing challenging and complicated structures (Quan et al., 2020). Hydrogels that photochemically crosslink and photocurable resins, such as shape-memory polymers or liquid crystal polymers, are a few of the materials that are often synthesized in four dimensions using SLA. The resin utilized for 3D/4D printing is frequently a shape-memory polymer that is designed to alter shape when subjected to a particular stimulus. A popular form of shape-memory polymer that is designed to alter shape by stimuli serves as one of the materials utilized in the 4D printing of scaffolds. These polymers have diverse qualities, including biocompatibility, biodegradability, and mechanical strength, and can be manufactured from several materials, including thermoplastics, thermosets, or elastomers. Hydrogels that are used to fabricate scaffolds replicate the extracellular matrix of tissues and are another material used for SLA-based 3D/4D printing of scaffolds. Natural polymers such as collagen, gelatin, and alginate, as well as synthetic polymers like PEG, are used to fabricate these hydrogels (Tamay et al., 2019). Overall, several materials are employed based on the purpose and expected attributes of the finished scaffold when employing the SLA process for 3D/4D printing scaffolds. SLA’s primary benefits are its superior surface quality and excellent printing resolution (Mitchell et al., 2018). Potential uses for SLA-based 3D/4D printing include the aerospace, biomedical, and robotics industries, all of which place a high value on products that may alter shape and conform to various environments.

#### 5.3.2 Stereolithography

Stereolithography (SL) is another 3D printing process that develops 3D objects by using a UV laser to cure liquid photopolymer resin. Photopolymerization of ceramic powder suspensions in monomer solutions begins the process, which is similar to gel casting. Along with photon dispersion in suspension, the characteristics of the monomer and photo-initiator are important in the photopolymerization technique. Photon dispersion in suspensions is composed of two components: particle diffusion and absorption by photo-initiators and inert dyes. The materials are fabricated by immersing a base in a liquid plastic monomer. Photopolymerization occurs when light rays interact with the top surface of a liquid. A second, thin layer of liquid monomer is put on top of a solid layer. The process is continued until the construction is completed (Pham and Ji, 2000). SLA is used in the process of 4D printing scaffolds to produce scaffolds with intricate shapes and microstructures that resemble the ECM of living tissue. The scaffold design is integrated with stimuli-responsive materials as part of the 4D printing technique so that they can alter shape or function according to reaction to external stimuli. Scaffolds are frequently printed using SLA with the following materials: photopolymer resins, polycaprolactone, hydrogels, ceramic, and metallic powders. However, the material for a scaffold that is printed using 4D technology must meet the scaffold’s required mechanical and biological qualities (Ahmed et al., 2021).

For pharmaceutical applications, AM techniques, particularly those utilizing a photo-curable process, offer significant benefits. For instance, these techniques present high-resolution capabilities, enabling the precise manufacturing of complicated structures. The absence of a nozzle in such practices reduces the risk of clogging or nozzle-related difficulties and increases the reliability of the production process. In addition, to maintaining material integrity and avoiding heat degradation, AM techniques in otolaryngology provide benefits such as increased layer integration by removing layer connection issues. However, to optimize their implementation, it is critical to recognize and overcome the limits of these strategies. One such constraint is limited selection of polymers and polymeric biomaterials suitable for pharmaceutical applications, as the starting ingredients in these processes must be photocurable. Furthermore, the types of polymers that are approved for therapeutic applications are subject to regulatory constraints. Ultimately, post-curing processes are frequently required to improve the mechanical qualities and stability of developed objects. Despite these disadvantages, the benefits of high-resolution processing, nozzle-free operation, decreased heat stress, and enhanced layer connectivity make additive manufacturing techniques a viable option for pharmaceutical manufacturing.

## 6 Application of additive manufacturing in management of otolaryngology

Additive manufacturing enables the fabrication of anatomical models that replicate patient-specific characteristics. These models are essential surgical planning aids as they provide surgeons with a physical and visual depiction of detailed anatomical correlations. In the area of otolaryngology, which is associated with the surgical management of ear, nose, and throat ailments, additive manufacturing has demonstrated potential for several applications.

### 6.1 Application of 3D/4D bioprinting in otology

Patients with hereditary and chronic ear abnormalities frequently experience significant psychological stress because of the ear’s significant sociocultural, aesthetic, and functional importance. Table 2 throws light on some current developments for 3D/4D bioprinting in otology. Surgery may become simpler and patient-specific casts may someday be practical with the formation of 3D bioprinting technology (Jovic et al., 2020).

**TABLE 2:** Recent application of 3D/4D bioprinting in otolaryngology.

Anatomic region	3D bioprinting techniques	Polymeric materials used	Cell source	Animal model used in assessment	Implant position in human	References
Auricle	Multi-nozzle	GelMA, polyethylene oxide and PCL	auricular chondrocytes	Mice	Auricle	Zhou et al. (2018)
Auricle	Laser sintering	PCL	-	Implanted beneath the thymus-free rats’ skin	Auricle	Brennan et al. (2021)
Auricle	Extrusion-based bioprinting	PCL	adipose derived stem cells (ASCs)	Rat	Auricle	Jang et al. (2020)
			co-cultured with chondrocytes			
Auricle	Extrusion-based bioprinting	PCL	Chondrocytes	Mouse	Auricle	Yin et al. (2020)
Auricle	Digital near infrared photopolymerization	Gelatin methacrylate	Articular chondrocytes from newborn rat	Subcutaneous implantation in BALB/c mice	Auricle	Chen et al. (2020)

Zhou et al. investigated the first significant clinical advancement on a global scale in 2018 using a tissue-engineered ear composed of chondrocytes and polyglycolic acid/polylactic acid. In order to enlarge the skin, a tissue expander and extracted microtia cartilage was inserted. For, *in vitro* cartilage engineering, the isolated microtia chondrocytes have been multiplied and implanted into the ear scaffold. After increasing the number of cells from around 4.5 million to 450 million the cell suspension rapidly propagated across the scaffold after being seeded onto it. After 12 weeks, laser scanning examination indicated that the regenerated ear form resembled the initial scaffold shape by more than 90%, indicating specific geometry modulation of the designed cartilage by modifying the scaffold (Zhou et al., 2018).

**FIGURE 1:**
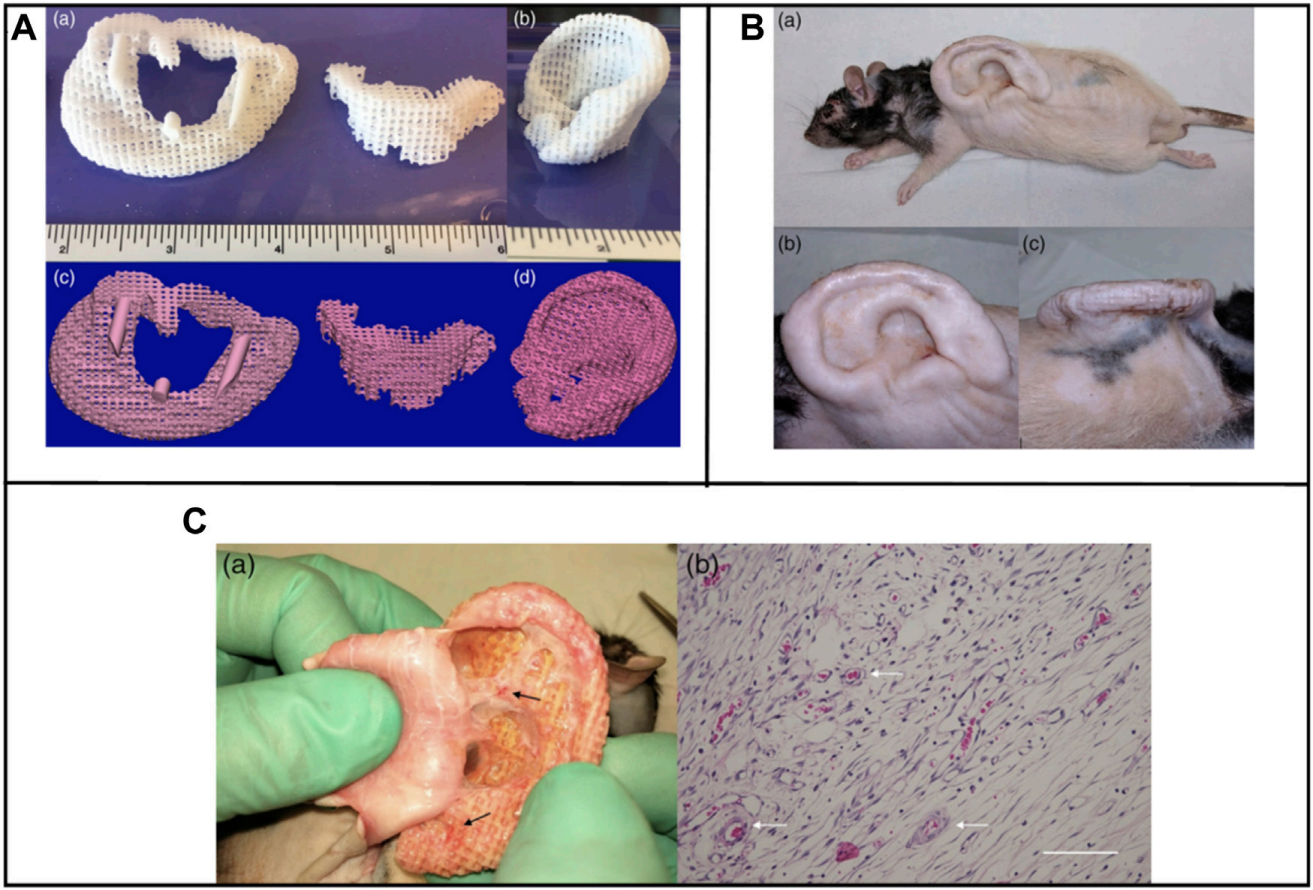
Lasser sintered 3D printing auricular cartilage scaffolds; laser sintered PCL two-stage, lock-in-key design **(Aa)**, laser sintered PCL single-stage design **(Ab)**, STL designed-image for two-stage, lock-in-key design **(Ac)**, STL, designed-image for single-stage **(Ad)**. Lateral view **(Ba, Bb)** demonstrating anatomic integrity of ear scaffold with side view demonstration **(Bc)**. Evidence showing angiogenesis upon harvesting the scaffold at 8 weeks **(Ca, Cb)**. Reproduce with permission from (Brennan et al., 2021) under right Links of John Wiley and Sons.

**FIGURE 2:**
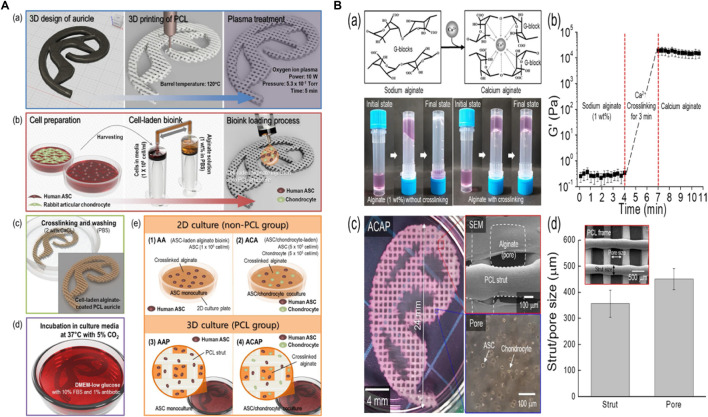
3D printing design and fabrication of PCL auricle structure **(Aa)**, preparation of cell-laden alginate solution and coating process **(Ab)**, crosslinking and rising processes **(Ac)**, and incubation for up to 28 days **(Ad)**. *In vitro* experimental groups such as ASC 2D culture in alginate (A1), ASC/chondrocyte 2D co-culture in alginate (A2), ASC 3D culture in alginate loaded on PCL structure (A3) and ASC/chondrocyte 3D co-culture in alginate loaded on PCL structure (A4). Calcium ion crosslinking of alginate and optical images showing viscose property before and after the crosslinking process **(Ba)**, rheological property before and after the cross-linking process **(Bb)**, optical and SEM images of cell-laden auricle scaffold **(Bc)** and strut/pore size analysis of the PCL auricle scaffold **(Bd)**. Reproduce with permission from (Jang et al., 2020) under right Links from ScienceDirect.

**FIGURE 3:**
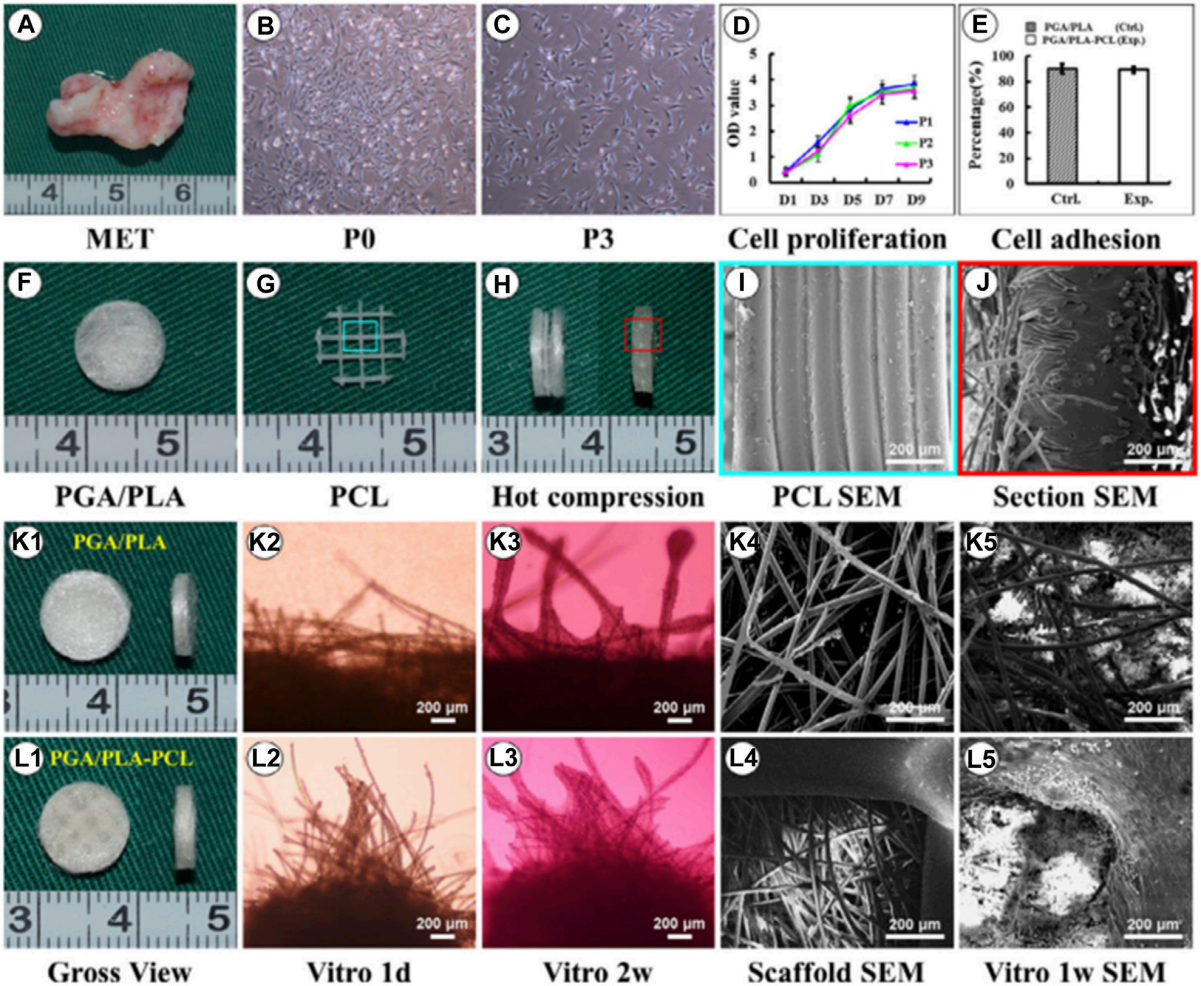
Biological performance of microtia chondrocytes on different scaffolds. Microtia ear tissue with an irregular shape **(A)**, microtia chondrocytes in P0 **(B)**, microtia chondrocytes in P3 **(C)**, indicate similar size and morphology. The cell growth of microtia chondrocytes reveals strong proliferation ability within 3 passages **(D)**. Fabrication of round-shaped PGA/PLA/-PCL scaffold **(F–H)**, scanning electron microscopy shows that after hot compressing, the multilayer structure of PCL **(I)** disappears and embeds with PGA fibers **(J)**. Gross view, optical observation, and scan electron microscopy show that microtia chondrocytes behave similarly in control group **(K1-K5)** and exp group **(L1-L5)**v with no significant difference in cell adhesion efficiency **(E)** after 24 h between the two groups. Reproduce with permission from (Yin et al., 2020) under Right Link share.

**FIGURE 4:**
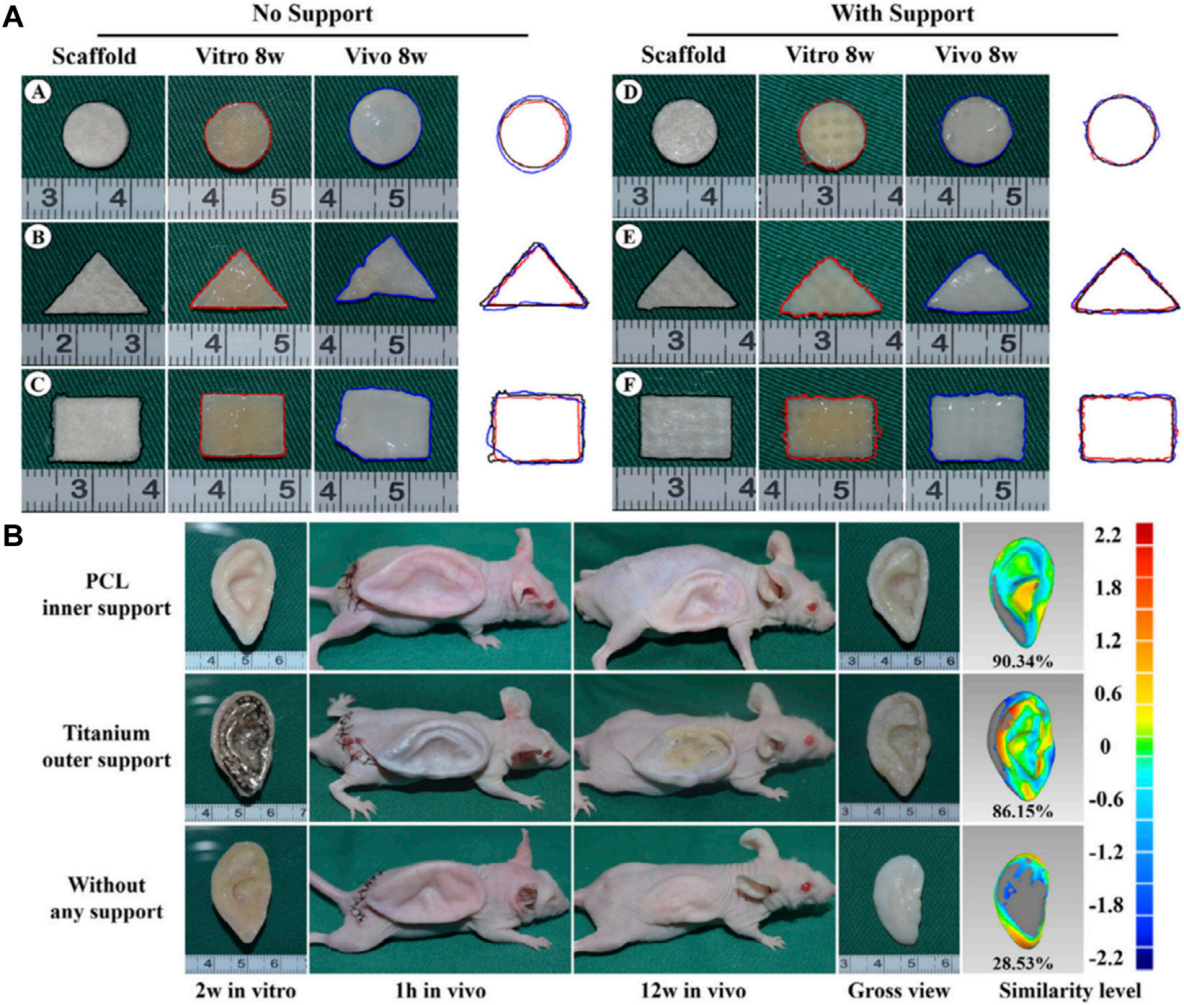
Gross observation and shape analysis of the constructs with different plane shape during different stages demonstrated that all groups maintain the original size after *in vitro* and *in vivo* incubation **(A**_**A-F**_ **)**. Furthermore, shape evaluation of ear-shaped construct with different support modes. After 2 weeks of *in vitro* culture, the auricular constructs are implanted in nude mice separately, including PCL inner support group, titanium outer support group, and without any support group indicated that all groups maintain their original auricular shape with detailed structures and high shape similarity of 90.34% and 86.15%, respectively. However, the specimen without any support almost loses its original shape and shrinks obviously with a shape similarity of 28.53% **(B)**. Reproduce with permission from (Yin et al., 2020) under Right Link share.

Brennan’s group examined an alternative way to fabricate 3D ear cartilage. Laser sintering of PCL was used for developing single- and two-stage auricular PCL structures. The scaffolds were placed under the skin of thymus-free animals. Implantation took an average of 22.4 min. The proportions of the ears maintained stable during the 8-week *in vivo* assessment, and no substantial dimensional contracture was seen regardless of the single-stage or two-stage designs. 67% of the 12 ulcers improved in the cross-sectional area, 25% worsened, and 8% remained steady. The scaffolds’ spherical pore design had 59% of porosity yet reduced total stiffness by more than 81%. According to the study, scaffold stiffness affected skin ulceration and dehiscence issues, with stiffer scaffolds causing difficulties due to more skin damage. At week 8, the stents were removed, and the results were analyzed by histologic staining and microcomputed tomography. In animal models, the auricular scaffold they developed and constructed exhibited great ease of implantation, appearance, vascularization, and tolerable superficial wound complication rates (Figure 1) (Brennan et al., 2021). To support the mechanical qualities of the regenerating auricle cartilage, Jang CH et al. designed a 3D hybrid scaffold by combining poly (caprolactone) along with a cell-rich alginate hydrogel including adipose-derived stem cells (ASCs) and chondrocytes. In a rat model, they examined the potential benefits of this 3D cell-filled auricle scaffold for cartilage tissue production. In both *in vitro* and *in vivo* trials, the inclusion of ASCs co-cultured along with chondrocytes increased chondrogenic differentiation and hastened cartilage regeneration. Cell viability and proliferation were evaluated, and all groups had comparable total cell counts. Adipose-derived stem cells 2D culture in alginate and ASC 3D culture in alginate loaded on PCL structure groups, on the other hand, had 100% ASCs, whereas the ASC/chondrocyte 2D co-culture in alginate and ASC/chondrocyte 3D co-culture in alginate loaded on PCL structure groups possessed approximately 50% ASCs owing to the 1:1 cell mixture proportion. Above all specimens had an initial cell viability of 90%–92%. Immunocytochemistry with aggrecan and osteopontin antibodies was used to assess chondrogenic and osteogenic differentiation. Compared to the 2D culture groups, the 3D culture groups had significantly higher values for osteogenic differentiation, with the AAP group having the most significant OPN staining results (Figure 2) (Jang et al., 2020). Yin et al. evaluated the survival and longer-term result of elastic cartilage regenerated with a specific human-ear form using enlarged microtia chondrocytes and a biodegradable scaffold augmented with PCL inner support in their work. Hot-compressing, pre-molding, and 3D printing methods were employed for manufacturing polyglycolic acid/polylactic acid scaffolds using or without using PCL interior support. The results show that each cartilage formed by the cells of each patient was remarkably consistent in both quantity and quality, indicating excellent repeatability and stability of the cartilage formation technique (Figures 3, 4) (Yin et al., 2020).

**FIGURE 5:**
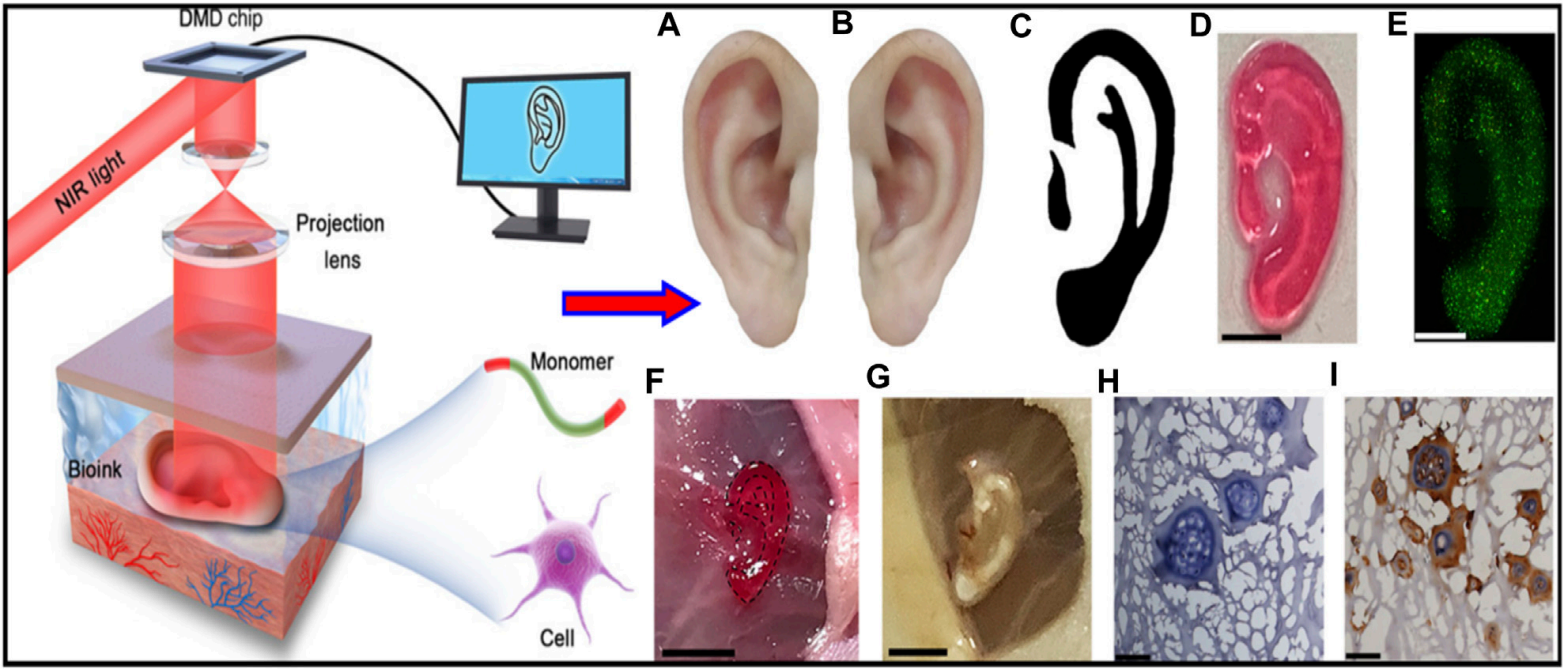
Illustrative representation of noninvasive 3D bioprinting technique using digital near-infrared photopolymerization. Non-invasive 3D bioprinting ear-like tissue by DNP process. Representative image of the normal ear **(A)**, mirror image of image **(A, B)**, optimized ear-outline image of **(B, C)**, image printed ear-like construct from the bioink covered over by skin by DNP process **(D)**. live/dead staining for ear constructs encapsulated with chondrocytes bioprinted using bioink covered by skin after cell culture for 7 days **(E)**, non-invasive 3D bioprinting of ear-shaped construct *in vivo* by DNP-process **(F)**, ear-shaped construct was printed subcutaneously in BALB/nude mice**(G, H, E, H)** and collagen type II immune staining of retrieved ear shape construct at 1 month **(I)**. Reproduce with permission from (Chen et al., 2020) under CC BY-NC.

**FIGURE 6:**
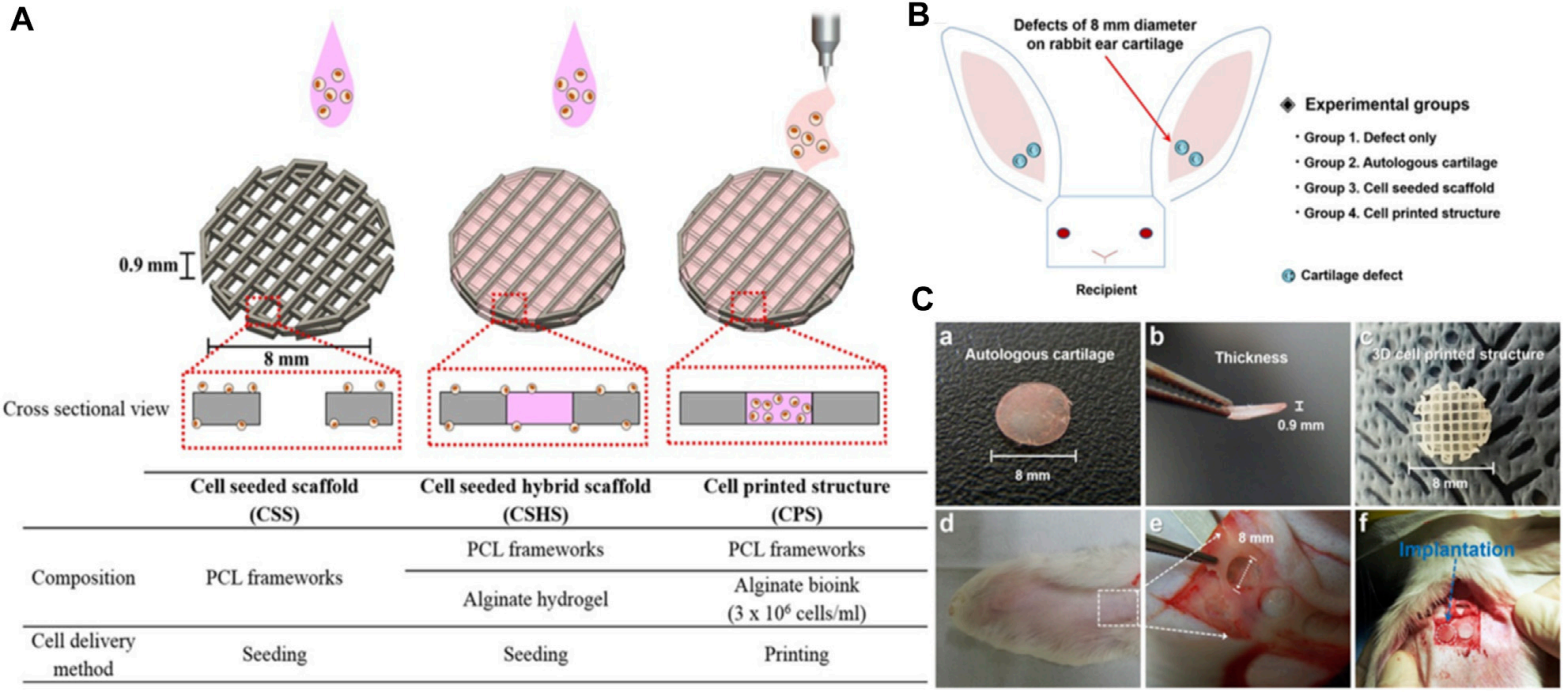
Schematic illustration of 3D printing of structure **(A)**. Illustration of animal experiments for an implant over rabbit ear **(B)**, autologs cartilage **(Ca-b)** cell printed structure **(Cc)**, defect site on the rabbit ear **(Cd)**, and defect creation using biopsy of 8 mm diameter **(Ce)**, implantation of the grafts into the cartilage defect of rabbit model **(Cf)**. Reproduce with permission from (Park et al., 2017) under Right Link share.

Chen et al. developed a non-invasive *in vivo* 3D bioprinting system, which employs digital NIR photopolymerization (DNP) 3D bioprinting to initiate the photopolymers crosslinking inside the body on the hypothesis that NIR could enter the tissues and allow precise control throughout crosslinking (Figure 5) (Chen et al., 2020). Gelatin methacrylate, a topically injected bioink, was crosslinked non-invasively via NIR photopolymerization, resulting in an ear-like scaffold (structures) inside the subcutaneous region of mice. The ear shape was maintained until 1 month, and histological examination revealed chondrocyte morphology with collagen type II synthesis, indicating hyaline cartilage. This research reinforces the applications of noninvasive *in vivo* 3D bioprinting technology for making complex tissues *in situ*. In another study, Park coworker indicated surgical technique using autologs cartilage is considered the best treatment for cartilage tissue reconstruction, although the burdens of donor site morbidity and surgical complication remain challenging. The effects on cartilage reconstruction were evaluated using 3D cell printing to fabricate tissue-engineered grafts. The cell viability and functionality of chondrocytes were significantly higher in cell printed structure, compared with cell seeded scaffold and cell-seeded hybrid scaffold *in vitro* indicating that cell printing technology can provide an appropriate environment in which encapsulated chondrocytes can survive and differentiate into cartilage tissue *in vivo.* Moreover, the effects of cell printing on cartilage regeneration were even better than those of auto logs cartilage (Figure 6) (Park et al., 2017).

### 6.2 Application of 3D/4D bioprinting in nasal reconstructions

**FIGURE 7:**
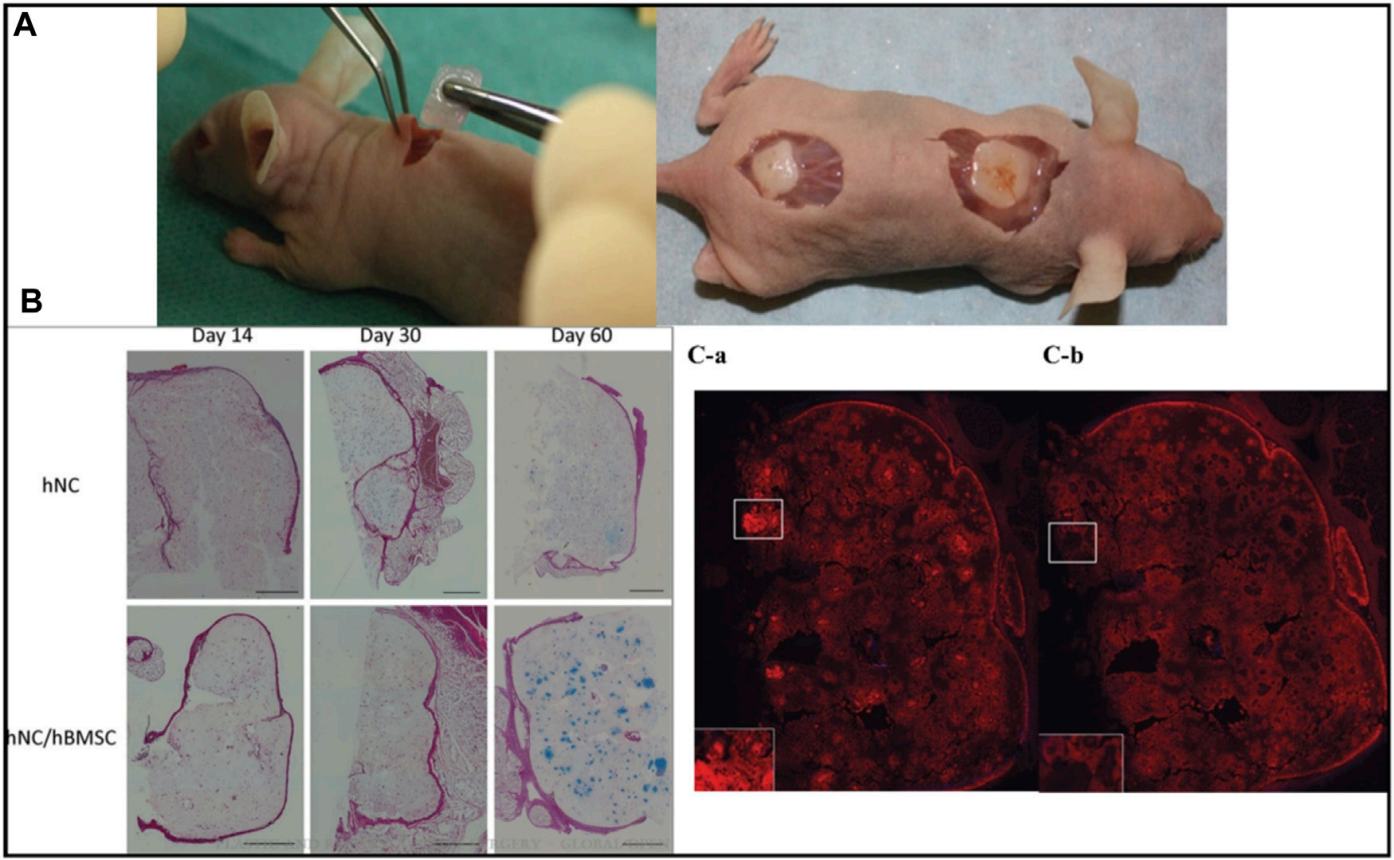
Photograph of human (male) nasal chondrocytes (hNC)-laden scaffold after 14 days of implantation. The constructs showed good handling properties **(A)**. The sample was surrounded by native mouse tissue and was well integrated **(A)**. Histological evaluation of GAG deposition over 60 days of implantation in hNC and hNC/human (female) bone marrow-derived mesenchymal stem cells (hBMSC) samples. Alcian Blue van Gieson staining was used to detect GAGs present in the newly synthesized ECM of day 14, day 30, and day 60 samples. Scale bars indicate 1,000 μm **(B)**. Immunohistochemical analysis of hNC/hBMSC group sample after 60 days of implantation. Samples were stained with mouse antihuman type II collagen antibody **(Ca)**, and mouse IgG antibody (isotype control) **(Cb)** to evaluate human type II collagen formation in the newly synthesized ECM. Scale bars indicate 1,000 µm. Reproduce with permission from (Möller et al., 2017) under Right Link share.

Nasal defects are major face deformities that result from facial tumors, infections, congenital illnesses, and trauma. As the use of 3D printing technology increased, doctors progressively discovered how useful it is to make customized prostheses and preoperative simulations before using it for nose reconstruction. Recent development in 3D/4D bioprinting for nasal reconstructions has been summarized in Table 3. Moller et al. designed 3D structures layer by layer utilizing polymeric biomaterials like biopolymers and cells. 36 female mice were implanted with a 5 × 5×1 (mm) piece of bio-printed cell-laden nano-fibrillated cellulose/alginate composite in a subcutaneous compartment. Even after 60 days of implantation, the scaffolds kept structural integrity and demonstrated favorable mechanical characteristics, according to the study. This shows that implants provide long-term tissue support and regeneration. Moreover, Histological and immunohistochemical analyses revealed that tissue integration was effective, although the mechanical properties of the scaffolds remained unchanged. These findings emphasize the scaffolds’ potential for tissue engineering applications (Figure 7) (Möller et al., 2017).

**TABLE 3:** Recent application of 3D/4D bioprinting in nasal reconstructions.

Anatomic region	3D bioprinting techniques	Polymeric materials used	Cell source	Animal model used in assessment	Implant position in human	References
Nasal cartilage	Fused filament fabrication method	cellulose/alginate	Human nasal chondrocytes and human bone marrow-derived mesenchymal stem cells	Mice	Nasal cartilage	Möller et al. (2017)
Nasal cartilage and subchondral bone reconstruction	fused deposition modeling	Gelatin/poly- (L-lactic acid)	Murine fibroblasts	*In-vitro*	Nasal cartilage and subchondral bone reconstruction	Rajzer et al. (2018)
Nasal cartilage	Fused filament fabrication method	Gelatin and Hyaluronic acid	Chondrocytes and ECM	Mice and goat	Nasal cartilage	Xia et al. (2018)
Nasal cartilage	Fused filament fabrication method	PCL	Human adipose-derived stem-cells hyalin cartilage-decellularized ECM pre-gel	Mice	Nasal cartilage	Yi et al. (2019)
Nasal cartilage	Projection based micro Stereolithography	PCL	Human nasal septal cartilage chondrocytes	Human	Nasal cartilage	Kim et al. (2018)

**FIGURE 8:**
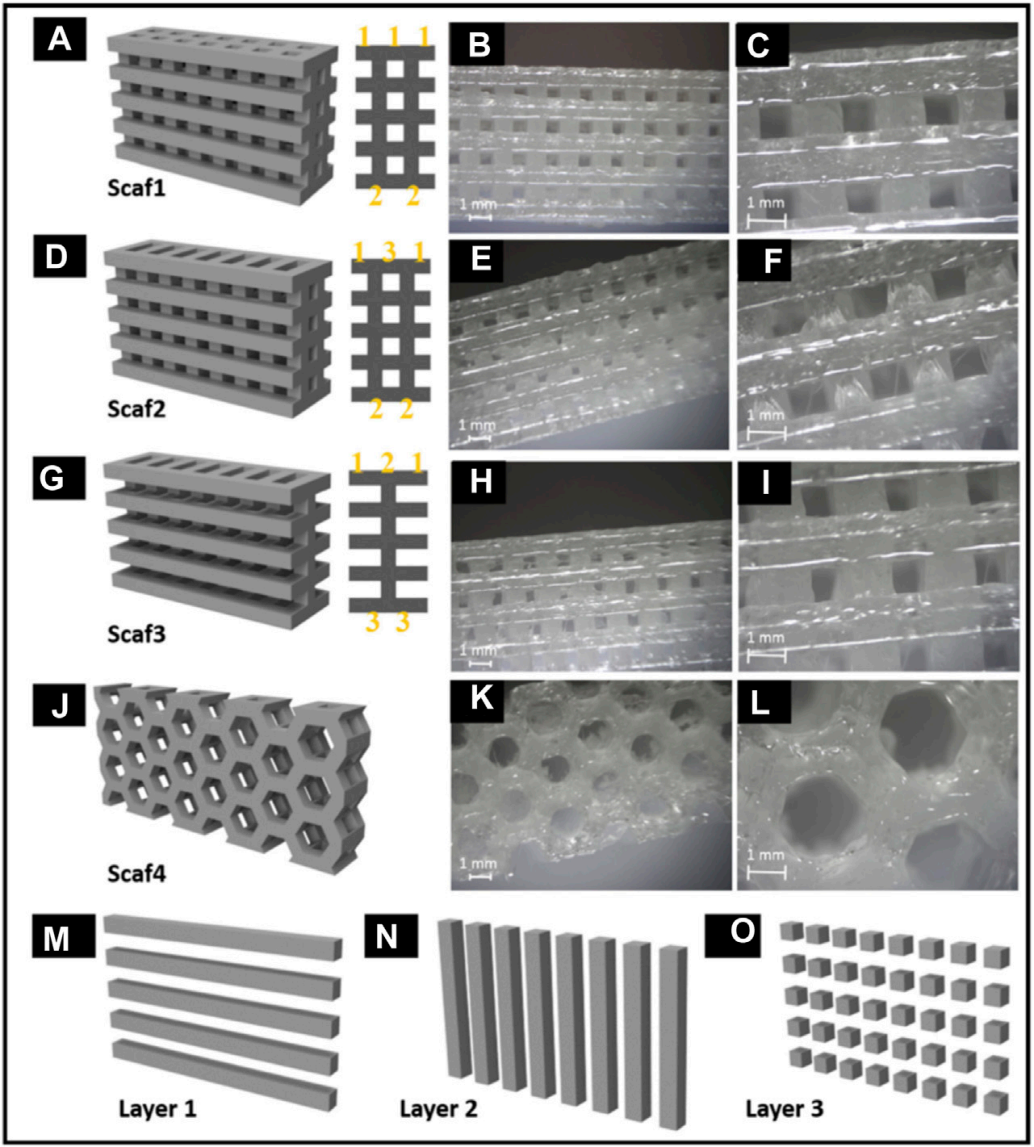
Multilayer gelatin/Poly-(L-lactic acid)/Osteo 3D printed scaffold for the reconstruction of subchondral bone and nasal cartilage **(A–O)**. Reproduce with permission from (Rajzer et al., 2018) under Right Link share.

Rajzer et al. developed a new versatile multilayer scaffold for nasal cartilage and subchondral bone replacement using biomaterials like Poly-(L-lactic acid) and gelatin using two scaffold manufacturing 3D printing processes, fused deposition modelling and electrospinning. The scaffolds fabricated using 3D printing technology were intended to alleviate the difficulty that otolaryngologists today have with attaching the nasal cartilage implant utilizing needles and threads. The multilayer gelatin/Poly-(L-lactic acid)/Osteo scaffold that resulted may be suitable for the reconstruction of subchondral bone and nasal cartilage (Figure 8) (Rajzer et al., 2018).

Xia et al. designed a unique natural 3D printed scaffold with excellent exterior structure, mechanical strength, immunogenicity, degradation rate, and pore structure for nasal cartilage regeneration using a novel scaffold-fabrication technique for native polymers (gelatin and hyaluronic). To overcome this issue, 3D printing is adequate for chondrocyte adhesion and proliferation, as well as subsequent cartilage repair. By modifying the filling rate parameters during 3D printing, the current work optimized the scaffold pore size. In comparison to the 30% and 70% groups, scaffolds with a 50% infill density exhibited greater cell transplant effectiveness and more homogenous cell dispersion. As a result, scaffolds made with this value were employed in the following studies.

**FIGURE 9:**
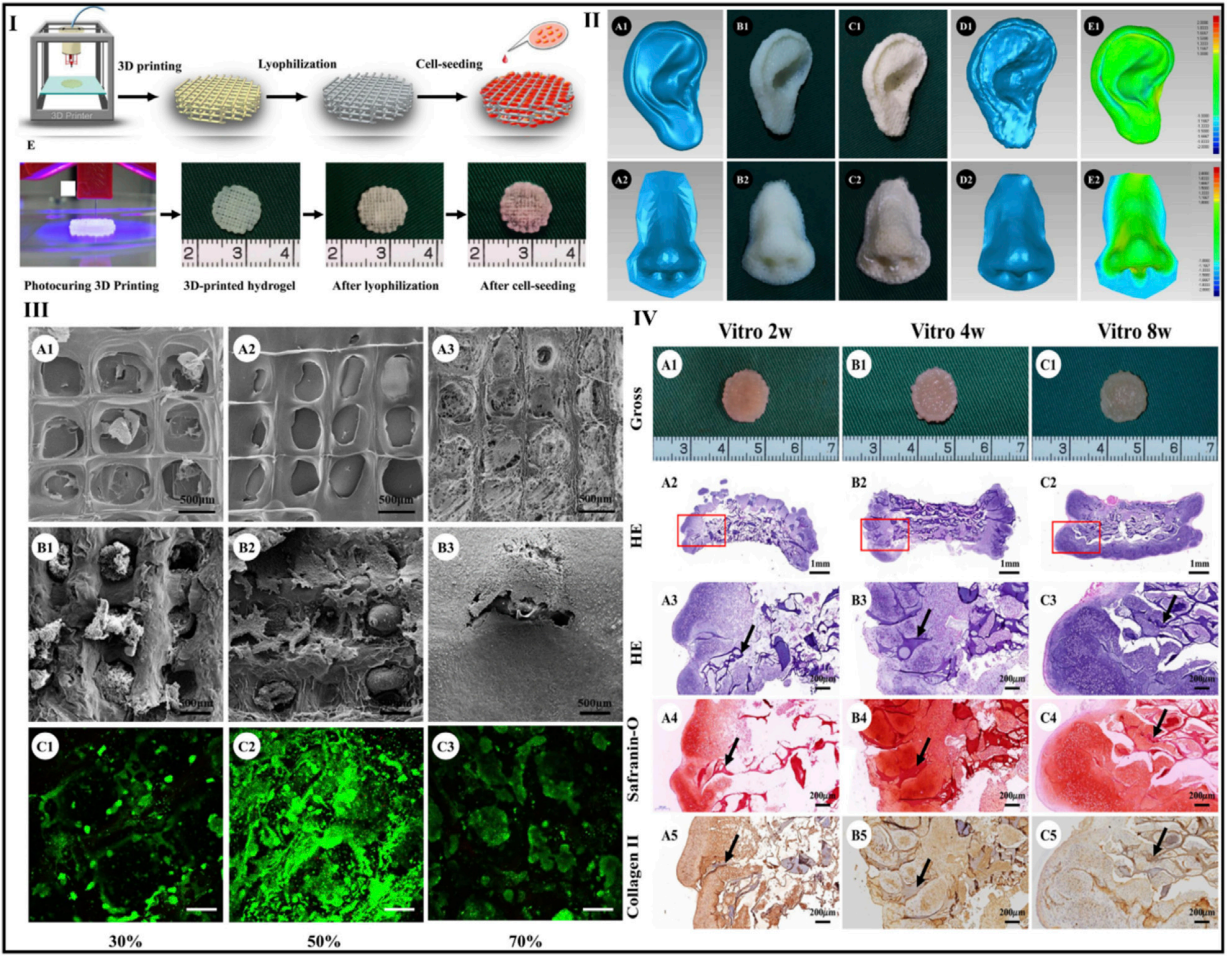
Scaffold preparation using photocuring 3D printing and lyophilization **(I)**. Human ear- and nose-shaped scaffolds and shape analysis: **(II**_**A1**_ **,**_**A2**_ **)** original digital models; **(II**_**B1, B2**_ **)** 3D-printed hydrogel; **(II**_**C1, C2**_ **)** scaffolds after lyophilization; **(II**_**D1, D2**_ **)** laser scan images of the lyophilized scaffolds; **(II**_**E1, E2**_ **)** shape similarity of the scaffolds compared with the digital models for ear- and nose-shaped scaffolds of 98% and 93%, respectively. The surface of the scaffolds presents different pore structures in the different groups with pore size significantly decreasing with increased infill density **(III**_**A1–A3**_ **)**. The scaffold in the 70% group fails to maintain an accurate pore structure **(III**_**A3**_ **)**. SEM **(III_B1–B3_)** and Live and Dead staining show that after cell seeding and 4 days of *in vitro* culture, pore structures in the 50% and 70% groups but not in 30% group were well-filled with chondrocytes and ECM **(III_C1–C3_)**. Gross view and histological examinations of *in vitro* engineered cartilage after cell seeding, samples at 2, 4, and 8 weeks retain their original shape and form cartilage-like tissues with a gradually matured cartilage appearance **(IV**_**A1–C1**_ **)**. Moreover, histologically, the engineered cartilage is preliminarily formed at 2 weeks with typical lacunae structures and cartilage-specific ECM deposition **(IV**_**A2–A5**_ **)** and matures with increased *in vitro* culture time accompanied by gradual degradation of the scaffolds **(IV_B2–B5_)** and **(IV_C2–C5_)** (black arrows indicate residual scaffold). Reproduce with permission from (Xia et al., 2018) under Right Link share.

Xia et al. combined gelatin and hyaluronic acid into a photo-cross-linkable hydrogel before 3D printing using a photocuring process to form scaffolds with accurate geometries and excellent interior pore designs. Furthermore, the hydrogel scaffolds were lyophilized. Most notably, tissue-engineered cartilage with characteristic lacunae structures and cartilage-specific ECM was effectively regenerated from chondrocyte-scaffold constructions *in vivo* and *in vitro*, showing a potential use of these scaffolds in the reconstruction of cartilage. Human nose- and ear-shaped hydrogels were effectively formed, and their lyophilized scaffolds increased maximum compressive strength (about 3-fold) and Young’s modulus (5-fold). At the same time, the *in vitro* degradation period was increased to about 8 weeks, which corresponded more closely to cartilage regeneration and it is observed that lyophilization increases the mechanical strength and reduces the breakdown rate of the 3D-printed hydrogel (Xia et al., 2018). Figure 9.

**FIGURE 10:**
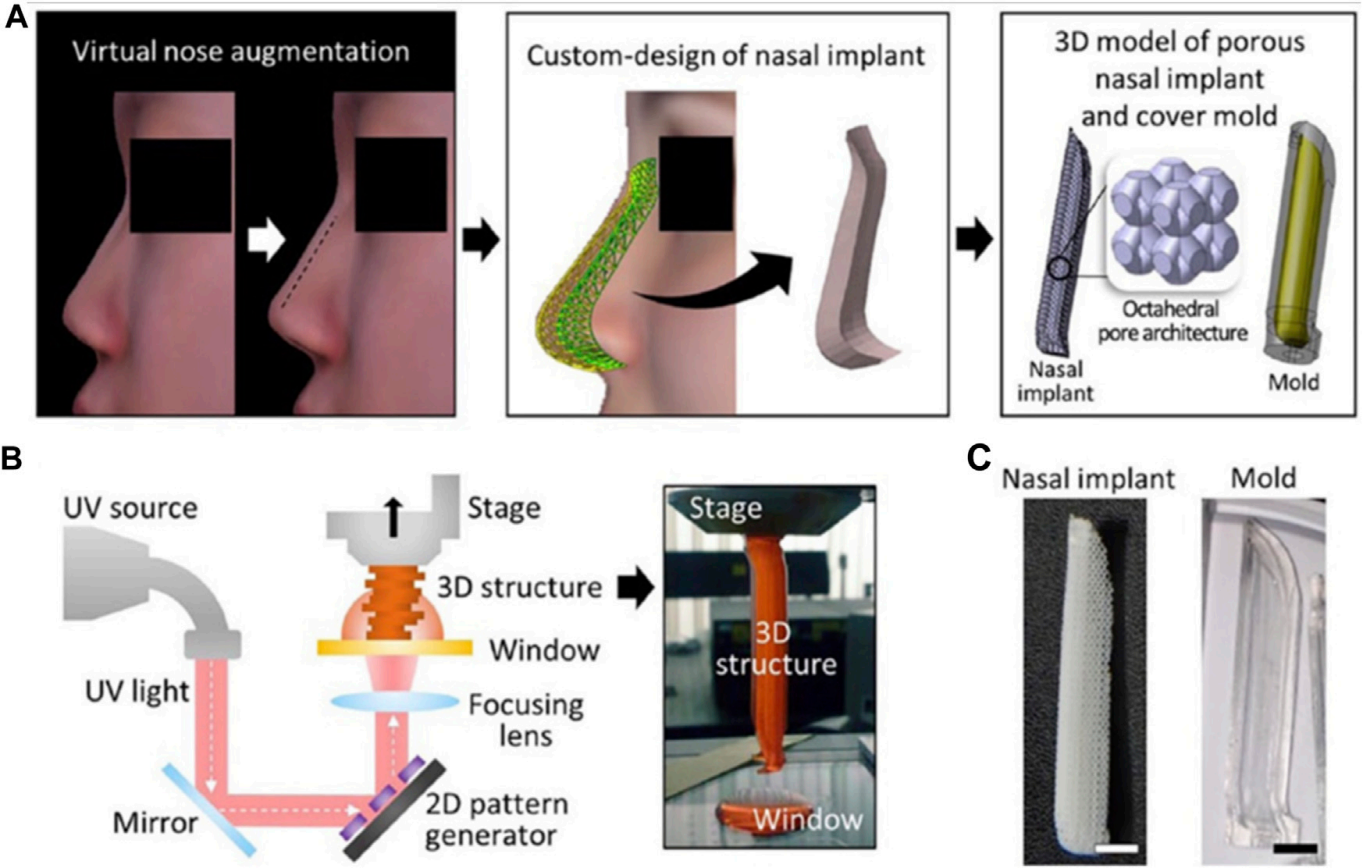
Computer-aided design and 3D printing of a patient-customized nasal implant. The process of generating the custom design of the nasal implant model. The difference between the preoperative and postoperative nose geometrical shapes was calculated. A 3D solid model was then generated according to the geometric difference. Finally, an octahedral pattern architecture was designed in the nasal implant model, and a cover mould model was designed based on the nasal implant model **(A)**. Illustration elucidating the principle of fabricating a 3D construct by the pMSTL system **(B)**. The fabricated PCL nasal implant and OrmoComp cover mould with the patient-specific design **(C)**. Reproduce with permission from (Yi et al., 2019) under Creative Commons Attribution-Non-Commercial 4.0 License.

**FIGURE 11:**
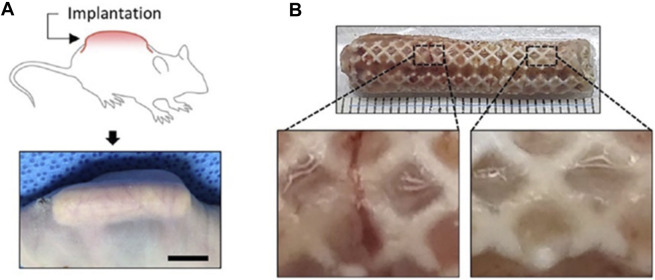
Subcutaneous implantation of the engineered nasal cartilage indicating construct implanted in a dorsal subcutaneous region **(A)** and gross image of the retrieved implant after 12 weeks post-implantation **(B)**. Reproduce with permission from (Yi et al., 2019) under Creative Commons Attribution-Non-Commercial 4.0 License.

Yi and colleagues integrated 3D printing with tissue engineering methods. Computer-aided design was used to form a 3D model of a personalized nasal implant in several phases. A cartilage-derived hydrogel containing human stem cells derived from adipose tissue was included in the implant’s octahedral internal layout to make a customized cartilage implant of nose and found that human adipose-derived stem cells expressed a high level of chondrogenic markers when cultured in synthetic nasal cartilage using cartilage-derived hydrogel. Additionally, the customized cartilage of the nasal maintained its structure and shape and demonstrated striking cartilaginous tissue formation for 12 weeks after being implanted into a mouse’s subcutaneous region (Figures 10, 11) (Yi et al., 2019)

**FIGURE 12:**
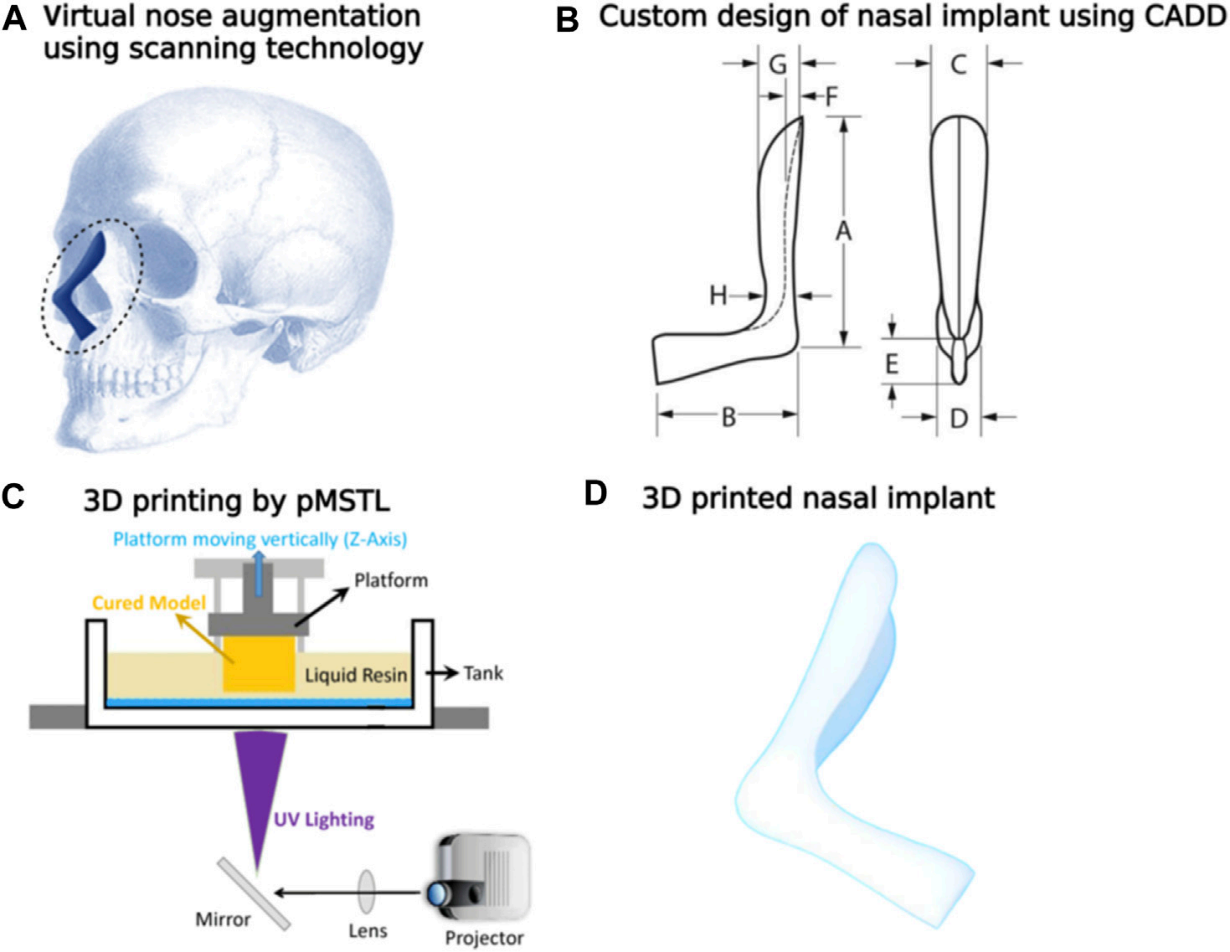
Virtual nose augmentation using scanning technology **(A)** using the computer-aided design, the nasal implant model was developed considering difference between the preoperative and postoperative nose geometrical shapes **(B)**. Diagrammatic depiction of a 3D construct by the pMSTL system **(C)**. Images of the fabricated PCL nasal implant **(D)**.

Kim et al. investigated the effectiveness and safety of 3-D printed, bioresorbable PCL nasal implants by using pMSTL system. This multidisciplinary clinical trial included 20 patients with caudal septal abnormalities who underwent septoplasty at 2 South Korean centers utilizing a 3D printed PCL mesh. The mechanical support, thinness, and surgical manipulability of the homogeneous, composite, microporous 3D printed PCL nasal implant were unique (Kim et al., 2018) as indicated in Figure 12. Augmentative rhinoplasty aims to increase the overall volume of the nose, with the pronasale as the centre.

### 6.3 Application of 3D/4D bioprinting in craniofacial bone reconstructions

The craniofacial tissues have a complex and finely organized 3D structure, consisting of 14 facial bones and 8 cranial bones. These skeletal components not only give rigidity and support but also act as a framework for the underlying soft tissues. The bones are comprised of an inorganic/organic matrix and result from intramembranous or endochondral ossification, according to histology. Mature bone is osteonal, with haversian systems and concentric lamellae of matrix containing osteocytic lacunae. On the other hand, the cartilaginous portion is made of chondroblasts and contains chondrocytes (Visscher et al., 2016). One of the most challenges, particularly in the restoration of function of craniofacial abnormalities, is to resemble these 3D intricate designs and multicellular interaction. Despite being the gold standard, autogenous grafts are not always readily available. Hence, bio-fabrication is the process of choice in recent time for producing craniofacial bone reconstructions utilizing bioprinters and live cells, along with special chemicals, extracellular matrices, and biomaterials (Table 4).

**TABLE 4:** Recent application of 3D/4D bioprinting in craniofacial bone reconstructions.

Anatomic region	3D/4D bioprinting techniques	Polymeric materials used	Cell source	Animal model used in assessment	Implant position in human	References
Calvarial bone	Laser assisted bioprinting	Polyether ketone	mesenchymal stem cells (MSC)	sheep	Calvarial bone	Adamzyk et al. (2016)
Mandible	Laser assisted bioprinting	Polyether ketone	Adipose-derived stem cells	Rabbit	Mandible	Roskies et al. (2017)
Calvarial bone	Laser sintering technique	Polyether ketone	Mesenchymal stem cells	Rabbit	Calvarial bone	Lin et al. (2019)
Craniofacial bone	Extrusion based bioprinting	polycaprolactone/hydroxyapatite	Adipose tissues	Mice	Craniofacial bone	Kuss et al. (2017)
Craniofacial bone	-	PCL and tricalcium phosphate along with Pluronic F127	hAFSCs	Rat	Craniofacial bone	Kang et al. (2016)

Adamzyk and co-worker investigated the survival, proliferation, and osteogenic growth of bone marrow-derived human and sheep mesenchymal stem cells (MSC) in conjunction with a 3D polyether ketone ketone (PEKK) (Anttiroiko et al., 2014) scaffold. For 12 weeks, they inserted cell-seeded 3D PEKK scaffolds into sheep with calvarial abnormalities to test if autologous MSC, either undifferentiated or osteogenically pre-differentiated, increased bone production following insertion. Assessment methods such as micro-computer tomography (micro-CT) and histological staining can be employed to evaluate the quantity and quality of the newly formed bone. The 3D PEKK scaffolds exhibit cyto- and biocompatibility, which facilitates adhesion, proliferation, and osteogenic differentiation of both human and ovine MSC (Adamzyk et al., 2016).

**FIGURE 13:**
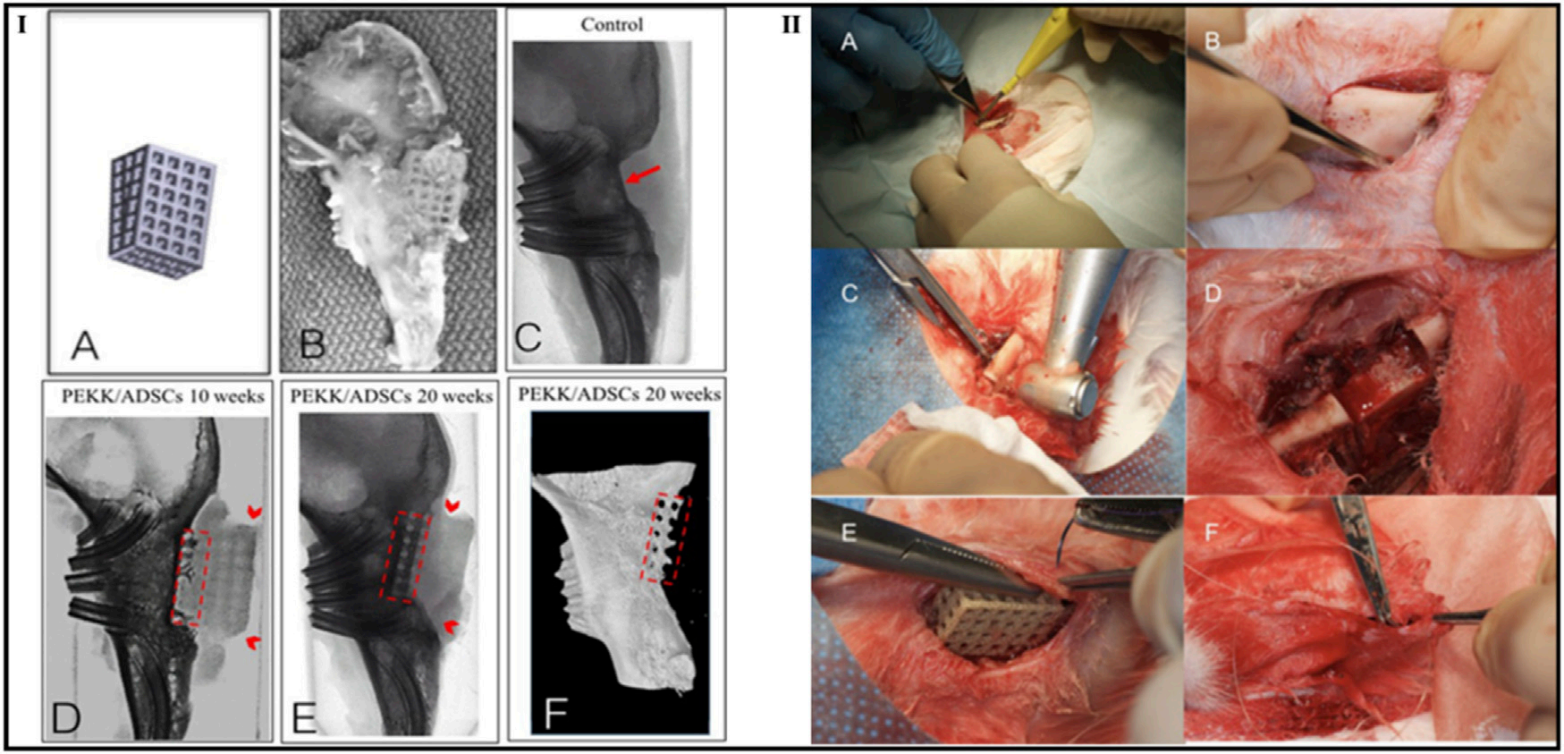
Computer-aided design image of a trapezoidal scaffold **(I**_**A**_ **)**, digital photograph of the PEKK/ADSCs composite at 10 weeks firmly embedded into adjacent bone **(I**_**B**_ **)**, microcomputed tomography images of a control group (without scaffold) at 20 weeks **(I_C–E_)** and **(I_C_)**, and PEKK/ADSCs group at 10 weeks **(I**_**D**_ **)** and 20 weeks **(I**_**E**_ **)**. The red arrow refers to the area lacking bone ingrowth in control critical-size bone defect while dashed red areas indicate the vertical bone ingrowth inside the radiolucent PEKK scaffolds. Additionally, red arrowheads refer to the margins of the PEKK scaffolds. Reconstructed microcomputed tomography 3D model showing bone integration in the PEKK/ADSCs group at 20 weeks (dashed red areas indicate the vertical bone ingrowth inside the PEKK scaffold). The second part of the figure demonstrates surgical procedure steps such as incision **(II_A_)**, exposure of mandibular body **(II**_**B**_ **)**, drilling of the bicortical critical-sized marginal bone defect **(II**_**C**_ **)**, mandibular defect **(II**_**D**_ **)**, placement of the PEKK/ADSCs composite scaffold **(II**_**E**_ **)**, and closure of the wound site with a musculofascial sling suture **(II**_**F**_ **)**. Reproduce with permission from (Roskies et al., 2017) under Right Link share.

Roskies et al. evaluated the capabilities of regeneration of the bone using 3D-printed PEKK scaffolds loaded with adipose-derived stem cells (ADSC) in a critical-sized bone defect developed from a rabbit model’s jaw. According to the study’s findings, mandibular lesions developed in an animal model can be filled in with osteointegration using a porous PEKK scaffold that was 3D printed and impregnated with ADSCs. Furthermore, a combination of radiologic, histologic, and morphological analyses supported the integration of the PEKK/ADSC composite in rabbit marginal mandibular critical-sized lesions. Each scaffold was well combined with the supporting bone. In the 10- and 20-week groups, bone-to-tissue volume expanded from 30.34 ± 12.46 to 61.27 ± 8.24 (%), and trabecular thickness expanded from 0.178 ± 0.069 to 0.331 ± 0.0306 (mm), respectively, compared to no bone regeneration on the control side. The integration of the bone-implant interface was verified by histology. Biomechanical tests found that the compressive resistance was 15 times greater than that of bone alone (Figure 13) (Roskies et al., 2017).

In the Lin et al. investigation, laser sintering was used for developing 3D-printed PEKK scaffolds. To assess their abilities for osteogenesis, proliferation, and cell attachment, human synovial fluid mesenchymal stem cells (hSF-MSCs) were characterized and grown on PEKK. To examine PEKK’s ability to regenerate bone when combined with hSF-MSCs, critical-sized bone abnormalities in the rabbit calvaria were produced. *In vitro*, research revealed that hSF-MSCs adhered to PEKK, multiplied, and were osteogenic. *In vivo,* studies demonstrate that PEKK seeded with osteogenically triggered hSF-MSCs regenerated twice as much newly made bone than PEKK scaffolds alone and observed that hSF-MSCs could be transplanted *in vivo* without initially being converted into osteoblasts. When PEKK and hSF-MSCs are utilized together, they may effectively regenerate critical-sized bone defects. The defects implanted with hSF-MSCs seeded on PEKK scaffolds (PEKK + SF) had the maximum amount of regenerated bone at 12 weeks, according to an *in vivo* study and found that after 12 weeks, PEKK seeded with hSF-MSCs had 20% bone volume compared to 9%–10% for the other 3 groups (p 0.05). Reconstructing the complicated structure of craniofacial abnormalities caused by cancer resection, trauma, and congenital malformations continues to be a challenging surgical process (Lin et al., 2019). Kuss et al. determined adipose tissue stromal vascular fraction and employed an endothelial cell medium to retain the shape and proliferation of lineage cells endothelium inside SVF-derived cells (SVFC). After 3D bioprinting SVFC using hydrogel bioinks the resulting structures were placed in hypoxia or normoxia. Despite long-lasting hypoxia, which decreased survival of cells and short-term hypoxia and vascularization increased the expression of vascularization-related genes. Athymic mice were implanted with 3D bio-printed bone constructs consisting of PCL/hydroxyapatite and SVFC-loaded hydrogel bioinks to assess their *in vivo* and *in vitro* vascularization and osteogenic differentiation.

Cooling in short-term hypoxic environments or nontoxic had previously been performed on the constructions. Short-term hypoxia developing increased micro-vessel development *in vitro* and *in vivo* as well as incorporation into the host vasculature, though osteogenic differentiation of SVFC was unaffected which suggests that SVFC combined with 3D bioprinting has the potential to produce pre-vascularized 3D bioprinted bone structures (Kuss et al., 2017). They also highlight the advantages of short-term hypoxia.

The advancement of 3D bioprinting technology could enable the development of custom-made implants for the reconstruction of maxillofacial abnormalities, particularly those caused by trauma, as well as the repair of head and neck cancer. Kang et al. fabricated not just cartilage scaffolds as well as a distinct 3D bioink that could be utilized to produce human-sized mandible bone parts. Human amniotic fluid stem cells (hAFSCs) had been combined with the bioink composed of mixture of composite hydrogel containing hyaluronic acid, glycerol, fibrinogen, and gelatin; and co-printing materials PCL and tricalcium phosphate along with Pluronic F127 as a temporary support. Using enhanced eosin/hematoxylin and tetra-chrome staining, the researchers discovered that the scaffold-designed novel vascularized bone tissue formed after *in vivo* implantation, in contrast to the undetected defect and scaffold-only treated control groups, which exhibited only minimal bone tissue formation and fibrotic tissue ingrowth. For future use of this technology, more research into its implementation in a mandibular deformity model is required.

### 6.4 Application of 3D/4D bioprinting in tracheal reconstruction

Tracheal reconstruction is an important medical therapy that is performed to fix or substitute a damaged or diseased trachea (windpipe). It is significant because it improves the quality of existence and general health of those who have tracheal deformities, stenosis (narrowing), or tracheal malignancy. 3D printing technology provides a viable way to overcome these obstacles and revolutionise tracheal repair. Based on unique anatomical data collected from medical imaging, 3D printing allows the construction of patient-specific tracheal structures (Table 5).

**TABLE 5:** Recent application of 3D/4D bioprinting in tracheal reconstructions.

Anatomic region	3D bioprinting techniques	Polymeric materials used	Cell source	Animal model used in assessment	Implant position in human	References
Trachea	-	polylactic acid	Chondrocytes	Rabbit	Trachea	Goldstein et al. (2015)
Trachea	-	polyurethane	Chondrocytes	Rabbit	Trachea	Jung et al. (2016)
Trachea	Extrusion-based Bioprinting	PCL and alginate	Nasal epithelial cells and auricular chondrocytes	Rabbit	Trachea	Park et al. (2019)
Trachea	Extrusion-based bioprinting	poly (l-lactic acid)	Rabbit auricular chondrocytes	Rabbit	Trachea	Gao et al. (2019)
Trachea	Stereolithography	Polycaprolactone	Human mesenchymal stem cells	Pig	Trachea	El-Husseiny et al. (2022)
Trachea	Digital light processing	Silk fibroin	Rabbit chondrocytes and TBSCs	Rabbit	Trachea	Rehmani et al. (2017)
Trachea	Electrospinning technology	Polycaprolactone	iPSC-derived mesenchymal stem cells, iPSC-derived chondrocytes, and human bronchial epithelial cells	Rabbit	Trachea	Kim et al. (2020a)

Goldstein et al. designs a graft for laryngotracheal repair (LTR) using 3D printing and tissue engineering. An anterior LTR transplant 3D computer model has been developed. A commercial 3D printer was used to produce the design using polylactic acid. The scaffolds were developed *in vitro* for up to 3 weeks after being seeded with mature chondrocytes and collagen gel. *In vitro*, tests were performed on scaffolds to measure cell viability and proliferation. On 9 white rabbits, newly developed scaffolds were utilized to conduct anterior graft LTR. At the four, eight, and 12-week marks, three animals were sacrificed. The *in vivo* transplant areas have been examined using both bronchoscopy and histology. *In vitro*, cell proliferation assay results showed 87.5% initial viability. Throughout the research period, the cells multiplied, doubling within the first week. Histology showed that the cells retained their cartilaginous properties during the 21-day experimental period. All animals survived the entire experimental period after *in vivo* testing. A well-mucosalized tracheal lumen without signs of scarring or granulation tissue was seen during bronchoscopy. Histological analysis further confirmed the existence of newly formed cartilage in the location where transplant was implanted (Goldstein et al., 2015).

Jung et al. developed a 3D-printed polyurethane tracheal scaffold that uses a micro-scale design enabling host tissue invasion and appropriate biomechanical characteristics to withstand physiological tracheal conditions. The 3D-printed tracheal scaffolds have been inserted into rabbits to test their *in vivo* function. Following implantation, tracheal scaffolds were found to be patent for 16 weeks after bronchoscopic examinations. According to histological studies, the cavity of the implanted scaffolds of the trachea re-epithelialized after 4 weeks of implantation and had ciliated epithelial cells associated with ciliary activity after 8 weeks of implantation. The connective tissue began to form the scaffolds 4 weeks after implantation. The implanted tracheal scaffolds’ biomechanical characteristics persisted throughout 16 weeks and show that treatment of partial tracheal anomalies using a 3D-printed tracheal scaffold could be an option (Jung et al., 2016).

Park et al. used 3D bio-printing to form an artificial trachea from autogenously separated epithelial cells and chondrocytes. The prosthetic trachea is effectively engrafted into partly resected tracheas, resulting in epithelialization and cartilage islet development. All rabbits displayed continual crackles or stridor following surgery. At euthanasia, the experimental group, as opposed to the control group, displayed higher respiratory scores. Radiographs revealed higher opacity at the implant locations in all rabbits. The control group exhibited a greater average drop in tracheal diameter ratio (46.19% ± 22.10%) than the experimental group (11.72% ± 13.81%). Particularly, the 12-month monitoring group showed a tracheal diameter reduction rate (6.72% ± 1.07%) comparable to that of a normal trachea. The experimental group survived until 12 months without respiratory indications, while the control group only survived four of six animals. The 3D-printed artificial graft made of autogenous cells can last for up to a year (Park et al., 2019). It is possible to develop a tissue-engineered trachea based on 3D-printed poly (l-lactic acid) scaffolds having a shape resembling a rabbit’s natural trachea for segmental tracheal repair.

The 3D-printed scaffolds were seeded with chondrocytes obtained from autologous auricula, dynamically pre-cultured *in vitro* for 2 weeks, and pre-vascularized *in vivo* for another 2 weeks. Then, segmental tracheal defects in rabbits were restored by transplanting the engineered tracheal substitute with pedicled muscular flaps. The combination of *in vitro* pre-culture and *in vivo* pre-vascularization generates a segmental tracheal substitute with mechanical properties and bionic structure identical to the native trachea of rabbits. Furthermore, the constant blood supply provided by the pedicled muscle flaps promotes chondrocyte survival and accelerates epithelialization, boosting survival rates (Gao et al., 2019).

Rehmani et al. set out to develop a method employing 3D printing to produce bioengineered tracheal grafts for the restoration of anterior tracheal lesions in a large-animal model (pig) that would have translational value for prospective human usage. Pigs with a 4 cm anterior lesion were implanted with a 3D-printed PCL graft, and it was shown that 5 out of 7 transplanted animals survived 90 days following transplantation (El-Husseiny et al., 2022).

**FIGURE 14:**
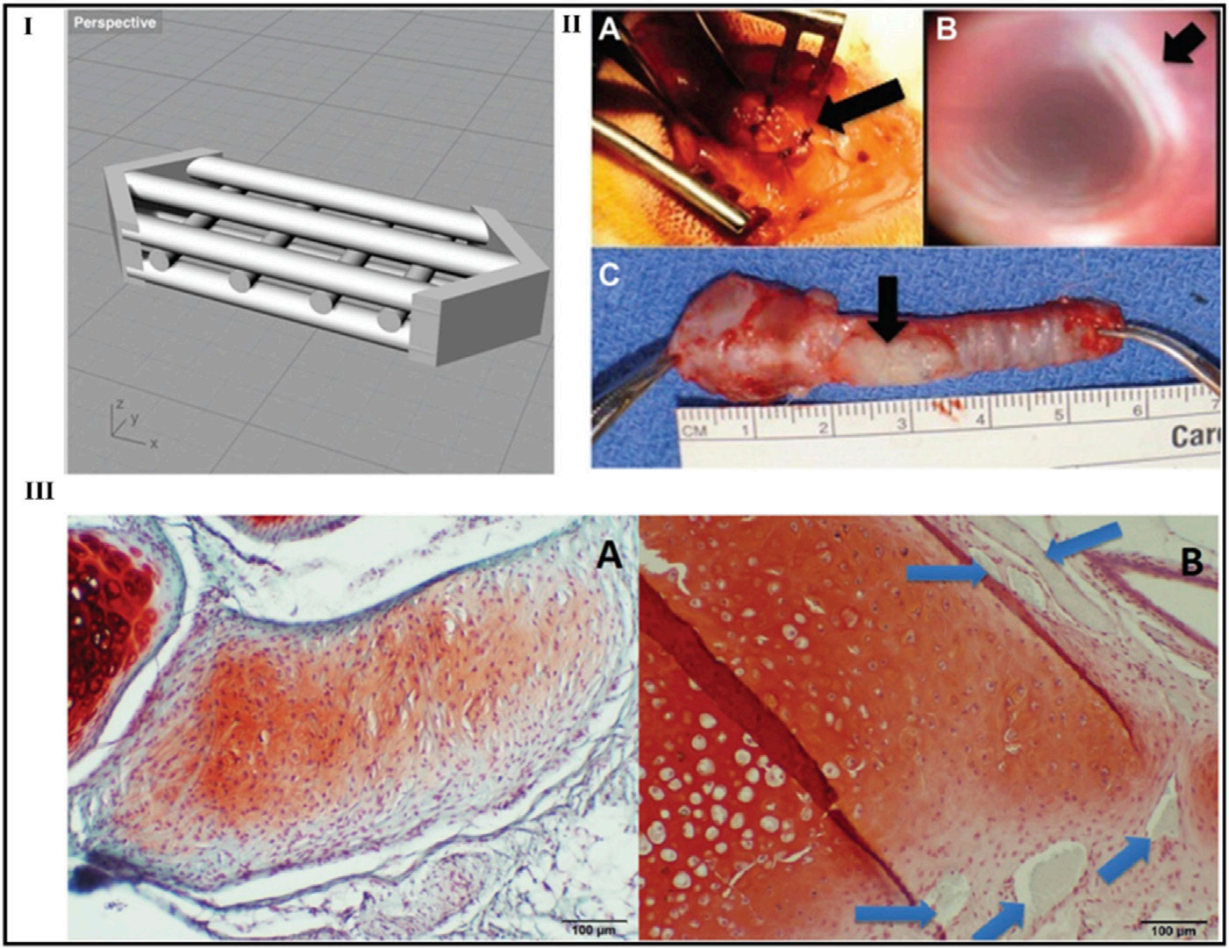
Laryngotracheal reconstruction scaffold design. Perspective view of the laryngotracheal reconstruction computer-aided design with multiple channels shown to allow for cellular incubation and growth **(I)**. Photographs of typical graft during and after *in vivo* placement in rabbit (Simonen et al., 2023): intraoperative view, **(II**_**B**_ **)** bronchoscopic view at 4 weeks, **(C)** *ex vivo* rabbit trachea with graft at 4 weeks. Arrows indicate the location of the graft **(II**_**A**_ **, II**_**C**_ **)** and the lumen of the trachea without granulation tissue or scarring **(B)**. *In vivo* histology: **(III**_**A**_ **)** *de novo* cartilage formation at 8 weeks and **(III**_**B**_ **)** neocartilage formed at 12 weeks. The original polylactic acid construct is still present (arrows). ×100 original magnification. Safranin O/fast green. Reproduce with permission from (Rehmani et al., 2017) under Creative Commons Attribution-Non-Commercial 4.0 License.

Biocompatibility and multi-component printability are lacking in 4D bioprinting. In addition, only theoretical and *in vitro* research has been done to develop appropriate implantable targets capable of using 4D bio-printed items. Kim et al. demonstrate a cellular-friendly and compatible 4D bioprinting method based on DLP and photocurable silk fibroin hydrogel. Bi-layered silk fibroin hydrogels that were 3D printed under physiological environments had their form alterations regulated by adjusting either their inner or exterior characteristics. performed finite element analysis models are being performed to investigate potential modifications in the intricate structure. This 4D bioprinting technology was used to fabricate trachea mimic tissue with 2 cell types and transplanted it for 8 weeks into a rabbit with a damaged trachea. Both epithelium and cartilage developed in the anticipated places after the implants spontaneously merged with the host trachea and show that the 4D bioprinting method can biologically form tissue mimic scaffolds, indicating the method’s potential for tissue engineering and therapeutic applications (Figure 14) (Rehmani et al., 2017).

**FIGURE 15:**
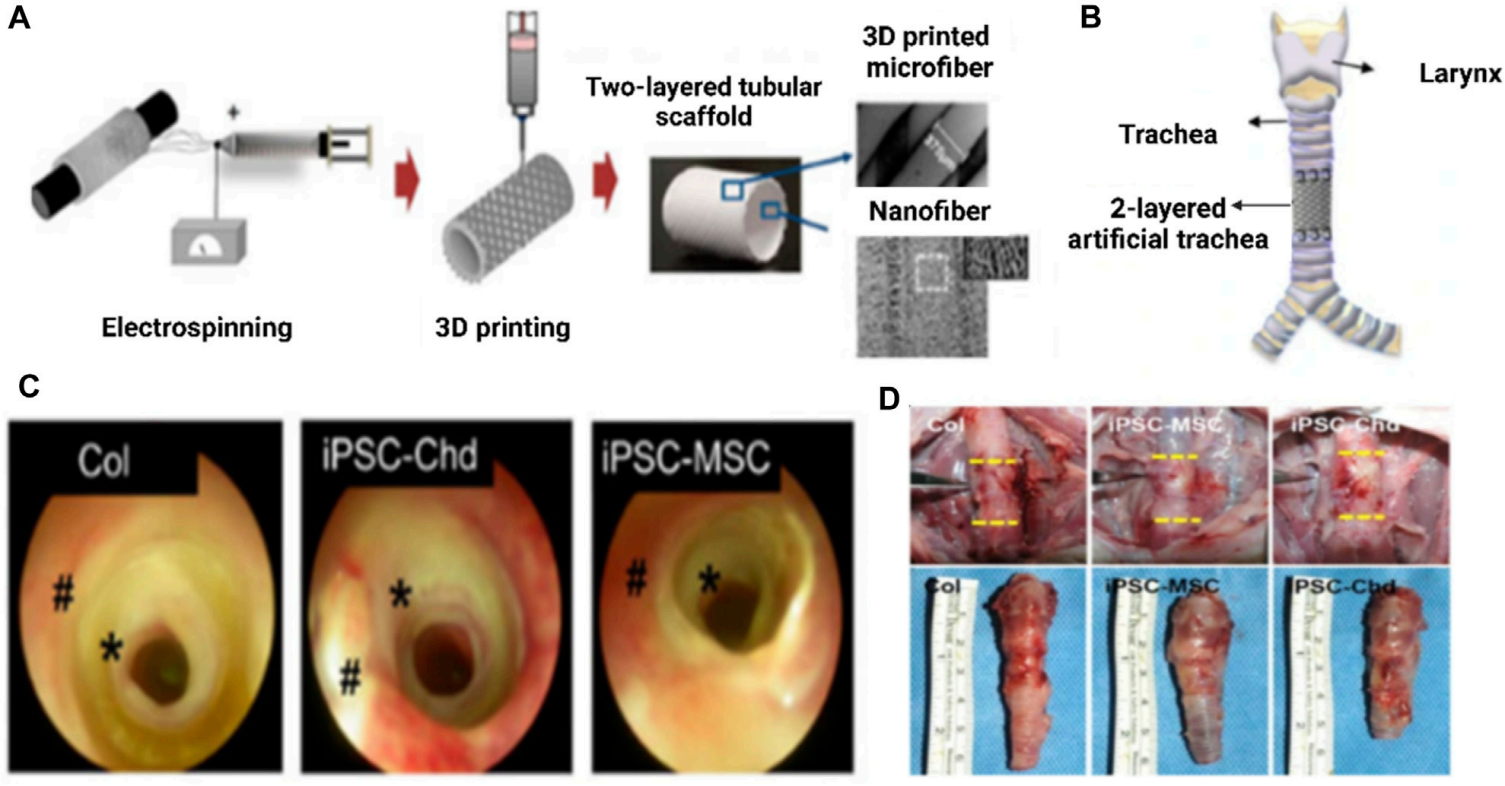
Tracheal transplantation and endoscopic analysis. Figure depicting the preparation process of two-layered tubular scaffolds by electrospinning 3D printing in 360° circumferential defects of rabbit trachea **(A)**, End-to-end implantation of artificial tracheal scaffolds in segmental tracheal defects **(B)**, Endoscopic images taken after 4 weeks reveals that airway patency was well-maintained in all three groups **(C)**, Macroscopic view of the harvesting of tracheal tissue at implantation site after 4 weeks of post-operative **(D)**. Reproduce with permission from (Kim et al., 2020a) under CC BY.

Kim et al. developed a synthetic trachea out of 3D-printed PCL microfibers (Roblegg et al., 2009) and electrospun PCL nanofibers (Roblegg et al., 2009). Additionally, cartilage regeneration and tracheal mucosa *in vivo* have been enhanced using iPSC-derived mesenchymal stem cells (iPSC-MSCs), iPSC-derived chondrocytes (iPSC-Chds), and human bronchial epithelial cells (hBECs). Following 2 days of development in a bioreactor system, tissue-engineered prosthetic tracheas were implanted within a segmental trachea defect rabbit model. Endoscopy revealed no evidence of granulation ingrowth into the tracheal lumen. The development of ciliated columnar epithelium in iPSC-MSC groups was effectively demonstrated with Alcian blue staining. Additionally, micro-CT examination demonstrated that iPSC-Chd groups successfully formed neocartilage at defect locations. As a result, their research provides a viable method for over a long period functional restoration of a segmental tracheal lesion as shown in (Figure 15) (Kim et al., 2020a).

## 7 Clinical application of otolaryngology and their commercial success

Otologists often face challenges related to intricate pathologic states. In this context, the true 3D visualization provided by 3D printed models offers great potential as a tool for improved treatment planning. Although much of the otologic applications to date have been in surgical education of trainees, namely, in temporal bone dissection, 3D printing is increasingly being utilized to develop patient-specific 3D printed models for preoperative otologic surgical planning and simulation.

Surgical management of otolaryngology requires precise understanding, access, and replications of complex anatomy. 3D printed implants have gained popularity amongst otolaryngology or head and neck surgeons in recent years, as 3D printed models provide the surgeon with a customized fit considering variations in patent anatomy. Ro and colleagues and Prisman and colleagues investigated value of preoperative mandibular contouring in patients undergoing reconstructions of mandible with osseous free flap reconstruction. The investigator group demonstrated that preoperative contouring was beneficial in restoring the contour of mandible postoperatively (Ro et al., 2007; Prisman et al., 2014). Additionally, Ro and co-worker indicated that this approach decreased operative time and provided a reconstructive method for tumors that extended lateral to buccal soft tissues (Ro et al., 2007). Furthermore, an oromandibular reconstruction, a 3D printed versatile surgical platform (V-stand) has also been utilized, serving as template to provide an excellent means for accurate spatial positioning of fibular free flap during surgery (Reiser et al., 2015). Among common use of 3D printing in otolaryngology, cancer surgery related to head and neck is in reconstruction of bony defects, especially following mandibular resection. Using rapid prototyping, custom mandible titanium trays have been designed, printed, and implanted, followed by autogenous bone grafting (Singare et al., 2004; Cohen et al., 2009; Zhou et al., 2010). Satisfactory aesthetic results including symmetry and quality of contour were achieved among treated patients with no reported severe complications. The potential benefits of 3D printing in otolaryngology related to cancer improved surgical planning, decreased operative time, and more accurate reconstruction (Patel et al., 2011). Several 3D printed model including laser-sintered 3D model to aid surgery for recurrent cholesteatoma involving complex bony structures and soft tissues found optimal for surgical planning (Suzuki et al., 2010), physical model based on their patient’s preoperative CT scan used to stimulate tympanomastoidectomy for a complicated recurrent cholesteatoma (Rose et al., 2015). Additionally temporal bone models have also been 3D printed and used as beneficial adjuncts for preoperative planning and simulation for the repair of tegmen tympani defects (Ahmed et al., 2017).

Advanced imaging that reflects commercial success, preoperative planning, fabrication of implants with 3D printing has a growing potential in paediatric otolaryngology as the field involves all aspects of head and neck surgery including complex airway cases, surgical rehabilitation of hearing loss, and endoscopic sinus surgery. 3D printing in paediatric otolaryngology was initially employed by Zopf and a co-worker who developed a 3D model of the tracheobronchial tree of a patient with severe bronchomalacia allowed an opportunity to practice orientation and placement of 3D printed airway splint (Zopf et al., 2013). Moreover, Morrison and co-worker developed and implanted patient-specific 3D-printed external airway splints in three infants with severe tracheobronchomalacia, and these splints were able to accommodate airway growth while preventing external compression before being bio-resorbed over time. Investigated clinical trials demonstrated resolution of both pulmonary and extrapulmonary complications of tracheobronchomalacia following external airway splinting (Morrison et al., 2015).

For years, manufacturing businesses have used 3D printing to manufacture product prototypes. Currently, companies use 3D printing for industrial medical applications. These include the companies Helisys, Ultimateker, and Organovo which employ 3D printing to produce living human tissue. Revotek has successfully implanted 3D-printed arteries into simian test subjects in partnership with specialists at Sichuan University’s West China Hospital. In 30 rhesus monkeys, a 2cm segment of the abdominal artery had been substituted with a 3D-printed blood conduit, and the stem cell bioink was formed using the autologous adipose mesenchymal stem cells (ADSCs) of animal (Wang and Hunt, 2017). The printer is capable of producing 10cm blood arteries in approximately 2 minutes utilising a print head containing two nozzles (Davies, 2016). Poietis employs technologies from INSERM and the University of Bordeaux. In order to form bioprinted skin models and hair follicles, respectively, the company works with BASF and L'Oréal (New release by Cornette, 2016). The company’s main emphasis is D laser-assisted bioprinting technology. They can print 3D objects as small as individual cells using their NGB 17.03 bioprinting device, which features an eight-axis motion. The Poieskin^®^ model, constructed with Poietis’ NGB bioprinter, was the first human full-skin model to be bioprinted (Poietis, 2018). 3D Bioprinting Solutions (3dbio) successfully 3D printed the world’s first animal thyroid gland in March 2015, and it was subsequently transplanted into a living mouse. Furthermore, 3D bio has partnered with Russia’s national space agency, United Rocket, and Space Corporation (URSC), to create artificial tissues aboard the International Space Station utilising a magnetic 3D bioprinter. Utilising this method, the business expects to make synthetic thyroid and renal tissue (Choudhury et al., 2018).

Though the study and investigations on 3D/4D printing has been in progress in the last 2 decades from now, and many patents are also granted. Currently some clinical trials are going on in US and Europe zone (Table 6) and there are also some commercial success stories available to talk.

**TABLE 6:** Update on Clinical trials of 3D/4D bioprinting (Data collected from https://clinicaltrials.gov).

Clinical trial ID	Topic	Status	Sponsored by
NCT05273060	Regenerative medicine approach to nasal reconstruction	Recruiting	Mayo Clinic
NCT03348293	Safety study of 3D printing personalized biodegradable implant for breast reconstruction	Recruiting	Xijing
			Hospital
NCT04098146	Registry to collect data on patients undergoing segmental mandibular defect reconstruction following oral squamous cell carcinoma resection	Recruiting	AO Innovation Translation Center
NCT03607227	Continuous popliteal block for microvascular free flap reconstruction in ear, nose, and throat surgery	Completed	Region Skane
NCT02559050	Nasal reconstruction using a customized 3d-printed nasal stent for congenital arrhinia	Completed	Seoul St. Mary’s Hospital

## 8 Patents

3D printing has been recognized for its potential in surgical modelling. Surgeons are nowadays able to fabricate lab made 3D printed models of the surgical task and this model can be used to educate the entrepreneur, plan the surgical approach, and act as an intraoperative surgical guide. 3D printed medical models are being used now days for management of cardiac, orthopaedic, dental, otolaryngology, and craniofacial applications. Surgical models developed using 3D printed technology significantly reduces surgical time and decrease complications during process. For orthopaedic and craniofacial applications, 3D printed surgical guides are used intra-operatively to determine the optimal location for internal plates and screws. 3D printed surgical reconstructive plates can be manufactured, which are customized for the patients and specific to their surgical requirements. Moreover, drill guides can be printed in advance to assist the surgeon with regards to optimal orientation, location, and depth for reconstructive screws and these guides are becoming increasingly accurate and more useful as innovations in transparency and flexibility become available for 3D printing (Kaye et al., 2016b). Summarize recent patent details are presented in Table 7.

**TABLE 7:** Summarize patent details on otolaryngology-based products.

Patent details	Title and Description	References
EP 3 270 821 B1 (2018)	*Artificial tympanic membrane devices* **:** The invention is for a biocompatible artificial tympanic membrane device constructed of polyglycolic acid (PGA) or polylactic acid (PLA). The tympanic membrane of a patient can be fixed, replaced, or patched using these artificial tympanic membrane grafts as implants. Interlocking bilayer grafts can also be utilized to fill tympanic membrane perforations	Skylar-Scott Mark et al. (2020)
US20230021383A1 (2023)	*Spatiotemporal delivery system embedded in 3D printing* **:** A 3D printing system and associated compositions, as well as a method of employing such, are provided herein, which can manufacture a polymeric microfiber with embedded microspheres encapsulating an active agent with micron accuracy and high spatial and temporal resolution	Lee Chang (2023)
US20230010971A1 (2023)	*Scaffold For Nasal Tissue Engineering* **:** A tissue scaffold component comprised of a biocompatible polymeric material with a plurality of open voids structured to enable cell development is included in a nasal tissue implant for the reconstruction and tissue engineering of nasal tissue in an individual. By employing additive manufacturing to fabricate the scaffold, it is possible to make implants that match the anatomy of each patient. This ensures that the scaffold fits securely and does not trigger distress to the patient	Zopf and Hollister Scott (2023)
US20060224242A1 (2006)	*Craniofacial implant* **:** The invention describes a craniofacial implant comprised of biocompatible materials like titanium. The implant is meant to be customized to the anatomy of every individual patient and can be utilized for treating a range of craniofacial abnormalities	Swords and Noble Aaron (2006)
US10639175B2 (2016)	*Porous bidirectional bellowed tracheal reconstruction device* **:** An acellularized tissue matrix and one or more support structures form a part of the structure that fits the patient’s deficient passageway in implantable splinting devices for supporting passageway defects. Several pores are also present in the structural component. The implanted splinting device can be fitted around a patient’s trachea, esophagus, bronchi, and blood vessels. The implanted splinting device can also be designed for implantation between a patient’s esophagus and trachea	Hollister Scott and Green Glenn (2020)

## 9 Future prospects and challenges

In otolaryngology, bioprinting has promising future potential. Bioprinted tissues and organs might be utilized to replace damaged or diseased tissue and develop new and improved surgical methods. Examples include the reconstruction of damaged voice cords using bioprinted cartilage, the replacement of larynxes that have been damaged by cancer or other disorders, and the reconstruction of hearing bones in the middle ear using bioprinted bone. The effectiveness of tissue regeneration can be increased using bio-printed tissues. Bioprinted tissues are a practical alternative for patients who necessitate tissue regeneration since the utilization of patient-specific cells can lower the chance of rejection. Although bioprinting technology is still in its early stages, many medical institutions are unable to access it due to the printers’ high cost and complexity. The 3D printing tool is becoming increasingly important in the manufacturing of devices and systems in the disciplines of biomaterials and tissue engineering. It changed the biomaterials sector’s circumstances by designing specific patient devices with the appropriate organization and shape. Metals and polymers, for example, have frequently been utilized in the biomedical area as stimuli-responsive materials. 4D printing enables feasible, practical, dynamic, and responsive systems for tissue engineering applications by integrating material and responsiveness in a biomedical device. The utilization of 3D and 4D printing techniques is now promoting the development of new biomaterials and biomedical devices. The stimuli to which the materials are now susceptible are well-known but limited and hence it is still a challenging problem to develop various materials with multiple sensitivities for application in enhancing the dynamic nature of devices. Before being utilized in individuals, bio-printed tissues must pass strict regulatory standards, which might delay the advancement and usage of technology. The complex and distinctive structures of the ear, nose, and throat tissues make it challenging to recreate them using existing bioprinting methods.

Bioinks play a significant role in cell proliferation, notably in regenerative medicine and tissue engineering. Bioinks are specialized materials utilized for fabricating three-dimensional (3D) structures that can promote cell growth and organization, such as scaffolds or matrices. These specialized materials provide a favorable milieu that promotes cell adhesion, multiplication, and development. Bioinks provide a scaffold structure favorable to cell attachment by closely mimicking the native ECM. Bioinks can regulate cell behavior and promote the development of functional tissues by replicating the natural ECM and integrating bioactive chemicals. Bioinks’ intricate design and customization provide particular conditions for different cell types, optimizing proliferation within extensively built tissue structures. Before bioprinting can be utilized as a common therapeutic tool, many issues need to be resolved. Firstly, bioinks are still not as complex as natural tissues and frequently do not have the characteristics needed to sustain cell proliferation and differentiation. This is a significant challenge that must be overcome before bioprinting can become a standard therapeutic tool. Secondly regarding printing processes, current printing techniques cannot produce complicated structures precisely similar to real tissues. This is a challenge that must be overcome to bio-print organs and tissues that are equal to native tissues in terms of functionality. Finally, bio-printed tissues and organs must be safe and effective before they can be employed in clinical settings. This implies that a method must be developed to ensure that the bioinks are not harmful and that the printed tissues and organs do not cause unfavorable responses in patients. Bioprinting has the potential to revolutionize the way we treat otolaryngological illnesses and disorders with further study and development.

## 10 Conclusion

In otolaryngology, the rapidly developing discipline of 3D and 4D printing has great potential for altering the treatment of ear, nose, and throat ailments, as well as tissue regeneration. These technologies allow for the development of customizable biological materials with variable shapes and properties, developing the way for personalized medicine, smart pharmacology, and targeted therapeutics. However, the high cost and complexity of bioprinters restrict many medical organizations from gaining widespread access to this technology. Although these advances, significant difficulties remain before bioprinting may become a popular therapeutic tool. The review article discusses the various biomaterials and cells utilized in the fabrication of patient-centric 3D and 4D bio-printed objects, as well as the application of additive manufacturing in otolaryngology management.

## Abbreviations

3D, Three-dimensional; AM, Additive manufacturing; CAD, Computer-aided design; PCL, Poly (caprolactone); SLA, Selective Laser Assisted; ECM, Extracellular matrix; MSCs, Mesenchymal stem cells; SL, Stereolithography; FFFM, Fused Filament Fabrication Method; pMSTL, Projection-based Micro Stereolithography; PEKK, Polyether ketone ketone; ADSC, Adipose-derived mesenchymal stem cells; hSF-MSCs, Human synovial fluid mesenchymal stem cells; SVFC, Stromal vascular fraction derived cells; Hap, Hydroxyapatite; hAFSCs, Human amniotic fluid stem cells; LTR, Laryngotracheal repair; PU, Polyurethane; FEA, Finite Element Analysis.
